# Numerical investigation of dusty tri-hybrid Ellis rotating nanofluid flow and thermal transportation over a stretchable Riga plate

**DOI:** 10.1038/s41598-023-41141-1

**Published:** 2023-08-31

**Authors:** Humaira Sharif, Bagh Ali, Imran Siddique, Iqra Saman, Mohammed M. M. Jaradat, Mohammed Sallah

**Affiliations:** 1 Department of Mathematics, Government College University Faisalabad, Layyah Campus, Layyah, 31200 Pakistan; 2 School of Mechanical Engineering and Automation, Harbin Institute of Technology, Shenzhen, 518055 China; 3 Department of Mathematics, University of Management and Technology, Lahore, 54770 Pakistan; 4 Mathematics Program, Department of Mathematics, Statistics and Physics, College of Arts and Sciences, Qatar University, 2713 Doha, Qatar; 5 Applied Mathematical Physics Research Group, Physics Department, Faculty of Science, Mansoura University, Mansoura, 35516 Egypt; 6 Higher Institute of Engineering and Technology, New Damietta, Egypt

**Keywords:** Mathematics and computing, Nanoscience and technology, Physics

## Abstract

Due to high-ultra thermic significances, the nanosize materials are used in various chemical and mechanical engineering, modern technology and thermic engineering eras. For industrial growth of a country, one of the biggest challenges for engineers and scientists is improvement in thermal production and resources. In this study we analyzed the momentum and thermic aspects of MHD Ellis ternary nano material embedded with dust particles via stretchable Riga plate including volume concentration of dust material. The flow generating PDE’s for two phase models are minimized into dimensionless nonlinear ODE’s by using the right modification. To acquire the graphical results the BVP4c method was adopted in MATLAB software. Fundamental aspects affecting velocity and temperature have investigated through graphs. Additionally Nusselt number and skin friction have also been evaluated. Compared it with previous literature to check the validity of results. Finding reveals that as compared to dusty phase the performance of trihybrid nano phase thermal transport is improved. Moreover, the temperature profile increases for rotational and volume fraction dust particles parameter. Dusty fluids are used in numerous manufacturing and engineering sectors, like petroleum transport, car smoke emissions, caustic granules in mining and power plant pipes.

## Introduction

In heat transport system the application of nanomaterial play a fundamental role in different industrial procedures involving thermic and chemical operations. In numerous heat transport system, distinct liquids have been used as a thermal porters. Heat transport fluids are valuable to a various applications like automobile system^1,2^, heat transfer in power plants^3,4^ and system of temperature changing^5^. In heat transfer fluids the thermal conductivity plays a significant role on the performance of heat transport procedures and device performance. Heat transposition may be accomplished by using nanoliquids. Sharif et al.^6^ analyzed the energy effects on Eyring’s nanofluid with microorganisms. Hussain et al.^7^ investigated the Brownian motion impact in the presence of motile microorganisms. Nanofluids are produced by mixing micro size particles in base liquid like water, minerals, air etc. Although when more than one kind of nano materials are extant in base liquid, the nanoliquids is transferred into hybrid nanoliquids. Hybrid nanoliquids demonstrate an exceptional performance as compared to mono nanoliquids^8^. Therefore hybrid nanoliquids are widely used to enhance the better heat transport^9^. Timofeeva et al.^10^ demonstrated that the dynamic viscosity of alumina-based nanofluids varies with the geometry of the nanoparticles at different temperatures. Surface charge is linked to these variations in the agglomeration and interactions between each form of nanoparticle (platelets, bricks, blades, and cylinders) and the base fluid. This is in strong agreement with Sahu and Sarkar’s conclusion^11^, which states that Nanoparticle morphologies affect both the exergetic and energetic performance. Jiang et al.^12^ described the dynamics of nanofluids resulting from thermo-capillary convection created by various five nanoparticle forms (sphere, blade, brick, cylinder, and platelet). The amount of thermo-capillary convection was found to be at its highest in a nanofluid made of spherical nanoparticles, and at its lowest in platelet-shaped nanoparticles. Additionally, blade nanoparticles had a 22.8% Nusselt number increase, compared to a 2.8% rise in blade-shaped nanoparticles. Algehyne et al.^13^ reported numerically trihybrid nanoliquids flow using the concept of non-Fourier’s and diffusion factor. They revealed that as compared to single nanoliquids, hybrid and ternary nanoliquids have outstanding tendency for liquid energy and rate of velocity propagation. More studies on nano fluid flow subject to various geometries are cited in^14–17^.

In modern age, dusty fluid flow model has unique investigators interest because of its two phase system. This impact appears in liquids flow with the distribution of solid particles. For instance, the reaction of chemical through which droplets are generated with the dusty air velocity and consolidation of dusty particles in difficulties of fluidization. The significant former for planetary structure is constructed by mixing dust and gas particles known as cosmic dust. Many researchers utilized the model of dusty phase with boundary conditions and various flow structures. Therefore, the outcomes they provided are numerical and approximate approaches.First of all Saffman^18^ gave the idea of dusty fluid. By utilizing the theory of Stoke’s drag he derived the equations for dusty liquid. He also observed that heat transport rate increased by using suspending dusty particles. Ezzat et al.^19^ analyzed the dusty liquid transfer with free convection heat transport on a planar surface in the existing of porous media. Sivaraj and Kumar^20^ investigated the MHD unsteady dusty fluid along an irregular surface with variation of mass diffusion. Dey and Chutia^21^ presented the dusty nanoliquids flow with bio-convection past a vertically stretchable surface. Rehman et al.^22^ examined the dusty Casson nanofluid past a stretchable surface with magnetic field and Darcy forchheimer law. They observed that for higher values of time relaxation the energy profile decreases in both phases.

Knowledge about the rotatable fluid flow is very useful for mechanical engineering, radiators, chemical industry, bio-medical spin coating, centrifugal etc. They are utilized for rotatable machinery, devices of computer storage, lubrications and in various engineering field. Hussain et al.^23^ conducted research to overcome the unstable nanofluid magnetohydrodynamic flow through the permeable channel past the rotating device’s moving surface while accounting for mass and heat transfer. Khan et al.^24^ looked into the conformational entropy of bio-convection nanofluid flow between two stretchy rotating disks. Nazar et al.^25^ promoted flow difficulties with instability. Their findings indicate a smooth transition from the initial unsteady flow to the final smooth flow. Ali et al.^26^ deliberated the unsteady rotatable flux of a Maxwell fluid past a stretchable cylinder. Hussain et al.^27^ studied the Darcy -forchheimer nanoliquids flow past a rotatable disk. Liu et al.^28^ inquired the rotatable flux dynamics in frictional stir welding. More investigations on rotatable fluid flow subject to various geometries are cited in^29–32^.

Various applications of trihybrid dusty fluid in present technology, developed a motivation to formulate this article. The non-Newtonian dusty fluids have widespread applications in many engineering field and industries, like production of cement, nuclear reactors, thermal exchanger, petroleum extraction, pipe industry, metalworking, etc. By analyzing the aloft mentioned literature, we conclude that analysis on the two phase dusty trihybrid Ellis fluid through a rotatable Riga plate did not performed. The nonlinear problem is sort out via numerically by using BVP4c approach. The including parameters are drawn graphically, to investigate the fluctuation of various profiles. To investigate the variations in physical quantities the present outcomes have been compared with existing literature.

## Mathematical-formulation

**Figure 1:**
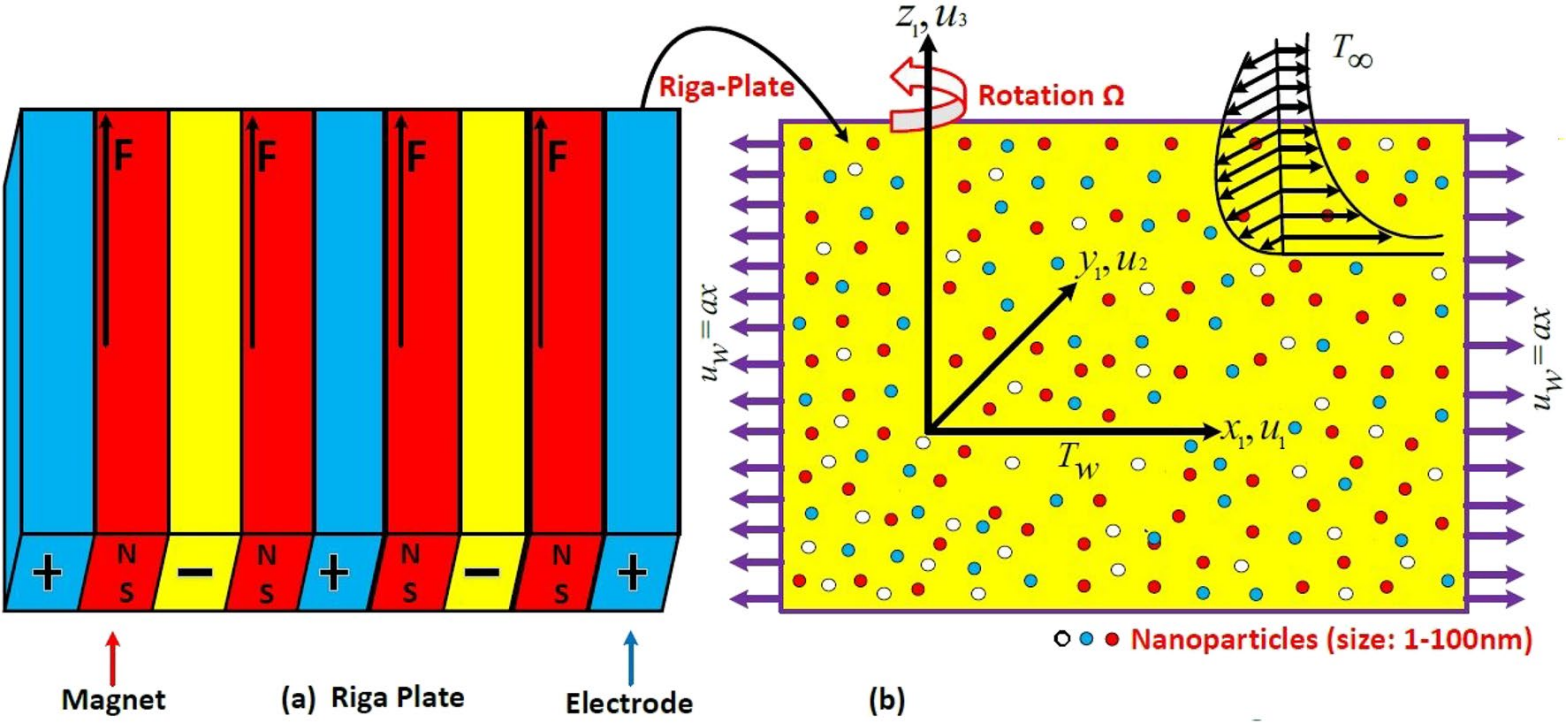
Flow model.

We assume steady, 3D rotatable of dusty tri-hybrid Ellis nanofluid flow by a stretching Riga plate. The combination of magnets and electrodes the Riga plate constructed. Due to electro-magnetic field of a Riga plate, force that is parallel to plate is Lorentz force. The plate ia stretchable in *xy* direction and liquid placed with the *z* direction. Along the *z*-direction the fluid rotate with $\Omega$ angular velocity. The velocity of primary flow is positive, the second body force is negative, that establish a negative effect on secondary velocity of fluid. Dust particles and fluid were assumed to be stable. The fluid is incompressible , therefore the dust particles density is constant and between the dust particles energy is prevent. Volume fraction of dusty particles has also been into account. Further, The plate having stretched velocity $U_w$ along x-axis. Due to tri-hybrid nanofluid is considered a stable mixture, therefore nano size particles agglomeration is ignored. zero velocity is assumed at ambient surafce. $T_w$ and $T_\infty$ are the wall and ambient temperature. The model is sketched in Fig. 1.

Considering the aloft conditions, the conservation of momentum and temperature equations can be mentioned as^33,34^:

1 $$\begin{aligned}{} & {} \frac{\partial u_1}{\partial x}+\frac{\partial u_2}{\partial y}+\frac{\partial u_3}{\partial z}= 0, \end{aligned}$$

2 $$\begin{aligned}{} & {} (1-\Phi _d)\rho _{Thnf}(u_1\frac{\partial u_1}{\partial x}+u_2\frac{\partial u_1}{\partial y}+u_3\frac{\partial u_1}{\partial z}-2\Omega u_2) = (1-\Phi _d)\mu _{Thnf}\frac{\partial ^2u_1}{\partial z^2}+\nonumber \\{} & {} (1-\Phi _d)\mu _{Thnf}\frac{\partial }{\partial z}\left[ \frac{1}{1+\left( \frac{\partial u_1}{\tau _0^2\partial z }\right) ^{\alpha -1}}\frac{\partial u_1}{\partial z}\right] +KL(u_1p-u_1)+\frac{\pi {j}_0M_0}{8}e^{-(\pi /a)z}, \end{aligned}$$

3 $$\begin{aligned}{} & {} (1-\Phi _d)\rho _{Thnf}(u_1\frac{\partial u_2}{\partial x}+u_2\frac{\partial u_2}{\partial y}+u_3\frac{\partial u_2}{\partial z}+2\Omega u_1) = (1-\Phi _d)\mu _{Thnf}\frac{\partial ^2u_2}{\partial z^2}+\nonumber \\{} & {} (1-\Phi _d)\mu _{Thnf}\frac{\partial }{\partial z}\left[ \frac{1}{1+\left( \frac{\partial u_2}{\tau _0^2\partial z }\right) ^{\alpha -1}}\frac{\partial u_2}{\partial z}\right] +KL(u_2p-u_2), \end{aligned}$$

4 $$\begin{aligned}{} & {} (\rho C_P)_{Thnf}(u_1\frac{\partial {T}}{\partial x}+ {u_2}\frac{\partial {T}}{\partial y}+{u_3}\frac{\partial {T}}{\partial z})={k}_{Thnf}\frac{\partial ^2{T}}{\partial z^2}+\frac{\rho _p C_p}{\tau _T}(T_p-T). \end{aligned}$$

For dusty phase

5 $$\begin{aligned}{} & {} \frac{\partial u_{1p}}{\partial x}+\frac{\partial u_{2p}}{\partial y}+\frac{\partial u_{3p}}{\partial z}= 0, \end{aligned}$$

6 $$\begin{aligned}{} & {} (u_{1p}\frac{\partial u_{1p}}{\partial x}+u_{2p}\frac{\partial u_{1p}}{\partial y}+u_{3p}\frac{\partial u_{1p}}{\partial z}-2\Omega u_{2p}) =\frac{KL}{\rho }(u_1-u_{1p}), \end{aligned}$$

7 $$\begin{aligned}{} & {} (u_{1p}\frac{\partial u_{2p}}{\partial x}+u_{2p}\frac{\partial u_{2p}}{\partial y}+u_{3p}\frac{\partial u_{2p}}{\partial z}+2\Omega u_{1p}) =\frac{K L}{\rho }(u_2-u_{2p}), \end{aligned}$$

8 $$\begin{aligned}{} & {} \rho _{p} c_p(u_{1p}\frac{\partial T_p}{\partial x}+u_{2p}\frac{\partial T_p}{\partial y}+u_{3p}\frac{\partial T_p}{\partial z})=\frac{\rho _p c_p}{\tau _T}(T-T_p). \end{aligned}$$

The appropriate boundary conditions are^35,36^:

9 $$\begin{aligned} u_1= & {} U_w=ax,\ u_2=u_3=0, \ T=T_w,\ as\ z=0, \end{aligned}$$

10 $$\begin{aligned} u_1p= & {} u_1=0, \ u_2p=u_2=0, \ {u_3p}\rightarrow {u_3}, \ {T}\rightarrow {T}_\infty , \ {T_p}\rightarrow {T}_\infty , \ as \ {z}\rightarrow \infty . \end{aligned}$$

Here, (${u_1},  \,\, {u_2},\,\, {u_3}$) are velocities component in (*x*,  *y*,  *z*) directions. Dust particles velocity components are represented by (${u_1p},\,\, {u_2p},\,\, {u_3p}$), $\Omega$ denotes the constant velocity, $U_w$ denotes the stretchable velocity component in *x*-direction, *a* is stretchable constant rate (*a* is positive), $\rho _{Thnf}$ is density tri-hybrid nanoliquid, $\rho _p$ is dust particles density, $C_p$ is dust particles concentration, *T* is liquid temperature, $T_p$ is dust particles temperature, $c_p$ is specific thermal capacity of liquid, $k_{Thnf}$ is thermic conductivity of tri-hybrid, $\tau _{T}$ is thermic stability time, *K* is constant of Stoke’s drag and *L* is micro-rotation factor.

## Rheological and thermophysical characteristics

The thermophysical characteristics of $TiO_2$, *MgO*, $COFe_2 O_4$ ternary hybrid nanoliquid are^37^:

1. Viscosity
  $$\begin{aligned} \mu _{Thnf} ={\mu _f}{(1-\Phi _1)^{-2.5} (1-\Phi _2)^{-2.5} (1-\Phi _3)^{-2.5}}, \end{aligned}$$
2. Density
  $$\begin{aligned} \rho _{Thnf} = \bigg [(1-\Phi _1)\bigg [(1-\Phi _2)\bigg ((1-\Phi _3)(\rho _f + \rho _3 \Phi _3)\bigg )+(\rho _2 \Phi _2) \bigg ] +\rho _1\Phi _1\bigg ], \end{aligned}$$
3. Heat capcity
  $$\begin{aligned} (\rho C_p)_{Thnf} = (1-\Phi _1)\bigg ((1-\Phi _2)\bigg [(1-\Phi _3)(\rho C_p)_f + (\rho C_p)_{s_3} \Phi _3 \bigg ]+(\rho C_p)_{s_2} \Phi _2 \bigg ) +(\rho C_p)_{s_1}\Phi _1, \end{aligned}$$
4. Thermal conductivity
  $$\begin{aligned} \frac{k_{nf}}{k_{f}} = \frac{k_3+2k_{nf}-2\Phi _3(k_{f}-k_3)}{k_3+2k_{nf}+\Phi _3(k_{f}-k_3)}, \\ \frac{k_{hnf}}{k_{nf}} = \frac{k_2+2k_{nf}-2\Phi _2(k_{nf}-k_2)}{k_2+2k_{nf}+\Phi _2(k_{nf}-k_2)},\\ \frac{k_{Thnf}}{k_{hnf}} = \frac{k_1+2k_{nf}-2\Phi _1(k_{hnf}-k_1)}{k_1+2k_{nf}+\Phi _1(k_{hnf}-k_1)}, \end{aligned}$$

**Table 1:** Thermophysical properties of nano size particles and water base fluid.

Physical properties	${\textrm{TiO}_{2}}$	MgO	${{\textrm{COFe}}_{2}{\textrm{O}}_{4}}$	${\textrm{H}}_{2}{\textrm{O}}$
$\rho$	4250.0	3560	4907	0997.1
$C_p$	686.2	955	700	4179
$\kappa$	8.9538	45	3.7	0.613

## Similarity conversion

We assumed the following appropriate transformation^38^.

11 $$\begin{aligned} \left. \begin{aligned} \xi = \sqrt{\frac{a}{\nu }}z,\ u_1= {a}x{H_1}'(\xi ),\ u_2=axH_2(\xi ),\ u_3= -(a\nu )^\frac{1}{2}H_1(\xi ),\\ \theta (\eta )({T}_w-{T}_\infty ) ={T}-{T}_\infty ,\\ \xi = \sqrt{\frac{a}{\nu }}z,\ u_{1p}= {a}x{H_{1p}}'(\xi ),\ u_{2p}=axH_{2p}(\xi ),\ u_{3p}= -(a\nu )^\frac{1}{2}H_{1p}(\xi ),\\ \theta _{p}(\eta )({T}_w-{T}_\infty ) ={T_P}-{T}_\infty .\\ \end{aligned}\right\} \end{aligned}$$

Equation (1) is identically satisfied. By utilizing the aloft mentioned transformations in Eqs. (2), (3), (4), (5), (6), (7), (8), (9) and (10), we get

12 $$\begin{aligned}{} & {} \frac{(1-\Phi _d)}{Z_1Z_2}\bigg (\frac{1+(2-\alpha )(B'_1H''_1)^{\alpha -1}}{(1+(B'_1H''_1)^{\alpha -1})^2}\bigg )H'''_1+(1-\Phi _d)(H_1 H''_1+ 2\beta H_2-H'^2_1)+\frac{\beta _v\gamma _v}{Z_2}(H'_{1p}-H'_1)+\frac{C}{Z_2}e^{-d\eta }=0, \end{aligned}$$

13 $$\begin{aligned}{} & {} \frac{(1-\Phi _d)}{Z_1Z_2}\bigg (\frac{1+(2-\alpha )(B'_1H''_1)^{\alpha -1}}{(1+(B'_1H''_1)^{\alpha -1})^2}\bigg )H''_2+(1-\Phi _d)(H_1 H'_2-2\beta H'_1-H'_1H_2) +\frac{\beta _v\gamma _v}{Z_2}(H_{2p}-H_2)=0, \end{aligned}$$

14 $$\begin{aligned}{} & {} \frac{1}{Pr}\frac{Z_4}{Z_5}\theta ''+H_1\theta '+\gamma _t\beta _t(\theta _p-\theta )=0, \end{aligned}$$

For the dusty case

15 $$\begin{aligned}{} & {} H'^2_{1p}-H_1p H''_1p-2\beta H_2p+\beta _v\gamma _v(H'_1p-H'_1)=0, \end{aligned}$$

16 $$\begin{aligned}{} & {} H'_{1p}H_{2p}-H_{1p}H'_{2p}+2\beta H'_{1p}+\beta _v \gamma _v(H_2p-H_2)=0, \end{aligned}$$

17 $$\begin{aligned}{} & {} H_{1p}\theta '_p+\beta _t\gamma _t(\theta -\theta _p)=0. \end{aligned}$$

Boundary conditions are

18 $$\begin{aligned} \left. \begin{aligned} H_1(0) = 0,\ H'_1(0) = 1,\ H_2(0)=0,\ \theta (0) = 1,\ at\ \xi =0,\\ H'_1(\infty )\rightarrow 0,\ H_2(\infty )\rightarrow 0,\ H'_{1p}(\infty )\rightarrow 0,\ H_{2p}(\infty )\rightarrow 0,\ \theta (\infty )\rightarrow 0,\ \\ \theta _p(\infty )\rightarrow 0,\ H_{1p}(\infty )\rightarrow H_1(\infty ),\ at\ \xi \rightarrow \infty . \end{aligned}\right\} \end{aligned}$$

Here, C is modified Hartman parameter, d is non-dimensional parameter, $\beta$ is rotation parameter, $\phi _d$ is concentration of dust particles, Pr is Prandtl factor, $B_1$ is fluid parameter, $\beta _t$ is thermal dust factor, $\gamma _t$ is specified thermal ratio, $\beta _v$ is velocity of fluid particles, $\gamma _v$ is dust particles mass concentration, Mathematically,

$$\begin{aligned} \beta =\frac{\Omega }{a},\ Pr=\frac{(\mu c_p)_f}{k_f},\ B_1=\frac{1}{\tau ^2_0}\sqrt{\frac{a(ax)^2}{\nu _f}},\alpha =\frac{\pi }{p}\sqrt{\frac{\nu _f}{a}},\ \beta _t=\frac{1}{a\tau _T},\gamma _t=\frac{c_p}{c_m},\ C=\frac{\pi }{\rho _f8a^2},\\ \beta _v=\frac{K}{am}, \gamma _v=\frac{Lm}{\rho },\ Z_1=\frac{\mu _(thnf)}{\mu _f},\ Z_2=\frac{\rho _(thnf)}{\rho _f},\ Z_4=\frac{k_(thnf)}{k_f},\ Z_5=\frac{(\rho C_p)_(thnf)}{(\rho C_p)_f},\ \end{aligned}$$

The physical quantities are Nusselt number and skin friction coefficient are defined as:

19 $$\begin{aligned} \left. \begin{aligned} {Cf}_x =\frac{\tau _(xz)}{U^2_w \rho _nf},\\ {Cf}_y =\frac{\tau _(yz)}{U^2_w \rho _nf},\\ Nu = \frac{xq_w}{({T}_w-{T}_\infty )k_f},\ \end{aligned}\right\} \end{aligned}$$

Where ${Cf}_x$, ${Cf}_y$ are skin friction coefficients along *x* and *y*-axis, Nu is Nusselt number. The non-dimensional form of Nusselt number and skin friction coefficient are as follows:

20 $$\begin{aligned} \left\{ \begin{aligned} Cf_x({Re_x})^{0.5}&= 1/Z_1 \bigg (\frac{H''_1(0)}{1+(B_1H''_1(0))^{\alpha -1}}\bigg ), Cf_y({Re_x})^{0.5} = 1/Z_1 \bigg (\frac{H'_2(0)}{1+(B_1H'_2(0))^{\alpha -1}}\bigg ),\\ Nu_x{Re_x}^{-0.5}&= -Z_4\theta '(0), \end{aligned} \right. \end{aligned}$$

Where ${Re_x}= \frac{xU_w}{\nu _f}$ is the Reynolds number.

## Solution method

The bvp4c technique is commonly used for solving the initial value problems. This technique is very stable and easy to implement.The non-linear Eqs. (12), (13), (14), (15), (16) and (17) with boundary conditions (18) are solved numerically by using bvp4c method in MATLAB environment. In this method, the system of differential Eqs. (12), (13), (14), (15), (16) and (17) is reduced to first order ODE’s.

$$\begin{aligned} H_1(\xi )= & {} y_1, H'_1(\xi )=y_2, H''_1(\xi )=y_3, \\ y_3'= & {} \frac{-Z_1Z_2}{(1-\Phi _d)}\bigg (\frac{(1+(B'_1y_3)^{\alpha -1})^2}{1+(2-\alpha )(B'_1y_3)^{\alpha -1}}\bigg )\bigg [(1-\Phi _d)(y_1 y_3+ 2\beta y_4-y^2_2)+\frac{\beta _v\gamma _v}{Z_2}(y_9-y_2)+\frac{C}{Z_2}e^{-d\eta }\bigg ]=0,\\ H'_2(\xi )= & {} y_5, \\ y_5'= & {} \frac{-Z_1Z_2}{(1-\Phi _d)}\bigg (\frac{(1+(B'_1y_3)^{\alpha -1})^2}{1+(2-\alpha )(B'_1y_3)^{\alpha -1}}\bigg )\bigg [(1-\Phi _d)(y_1 y_5- 2\beta y_2-y_2y_4)+\frac{\beta _v\gamma _v}{Z_2}(y_10-y_4)\bigg ]=0,\\ \theta '(\xi )= & {} y_7,\\ y_7'= & {} \frac{-PrZ_5}{Z_4}\bigg [y_1y_7+\gamma _t\beta _t(y_{11}-y_6)\bigg ]=0,\\ H'_{1p}= & {} y_9,\\ y_9'= & {} 1/y_8\bigg [y^2_9-2\beta y_4+\beta _v\gamma _v(y_9-y_2)\bigg ],\\ H_{2p}= & {} y_10,\\ y_{10}'= & {} 1/y_8\bigg [y_9y_{10}+2\beta y_9+\beta _v\gamma _v(y_{10}-y_4)\bigg ],\\ \theta _p= & {} y_{11},\\ y_{11}'= & {} -1/y_8\bigg [\beta _t\gamma _t(y_6-y_{11})\bigg ],\\ \end{aligned}$$

With the relevant conditions are:

$$\begin{aligned}{} & {} y_1(0)=0,\ y_2(0)=1,\ y_4(0)=0,\ y_6(0)=1,\\{} & {} y_2(\infty )\rightarrow 0,\ y_4(\infty )\rightarrow 0,\ y_6(\infty )\rightarrow 0,\ y_9(\infty )=\rightarrow 0,\ y_{10}(\infty )\rightarrow 0,\\{} & {} y_{11}(\infty )\rightarrow 0,\ y_8(\infty )\rightarrow y_1(0).\\ \end{aligned}$$

## Results and discussion

**Figure 2:**
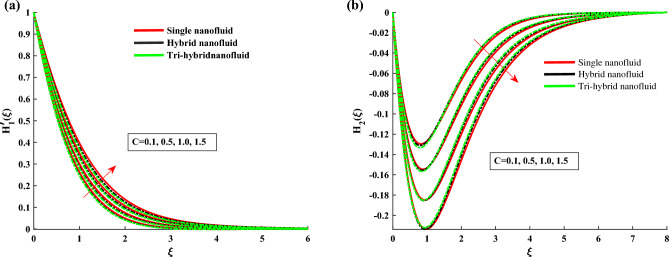
$H'_1$ and $H_2$ variation against *C*.

**Figure 3:**
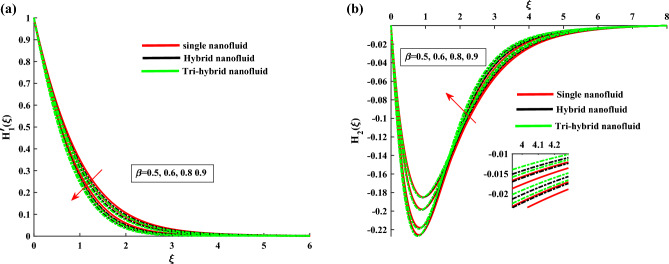
$H'_1$ and $H_2$ variation against $\beta$.

**Figure 4:**
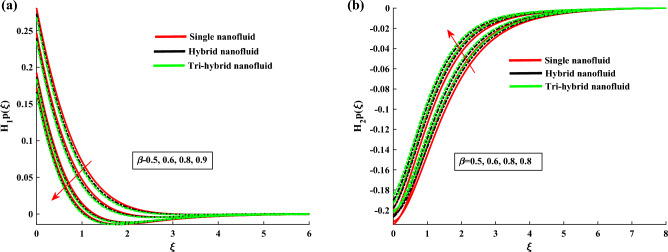
$H_{1p}$ and $H_{2p}$ variation against $\beta$.

**Figure 5:**
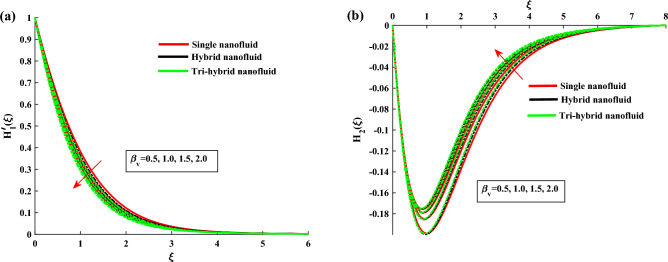
$H'_1$ and $H_2$ variation against $\beta _v$.

**Figure 6:**
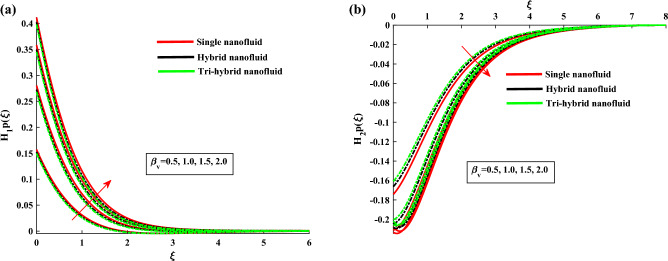
$H_{1p}$ and $H_{2p}$ variation against $\beta _v$.

**Figure 7:**
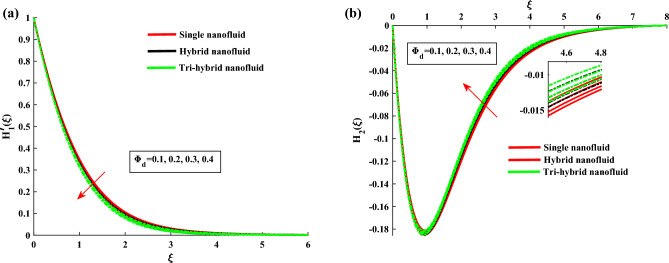
$H'_1$ and $H_2$ variation against $\phi _d$.

**Figure 8:**
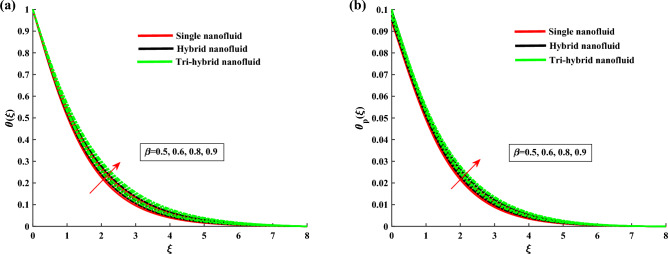
$\theta$ and $\theta _p$ variation against $\beta$.

**Figure 9:**
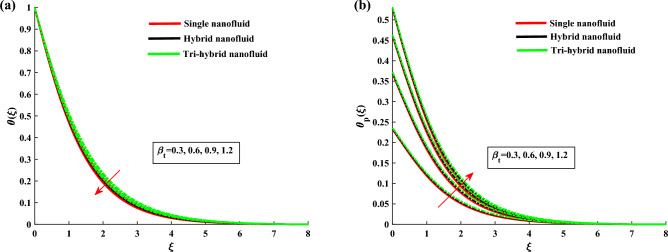
$\theta$ and $\theta _p$ variation against $\beta _t$.

**Figure 10:**
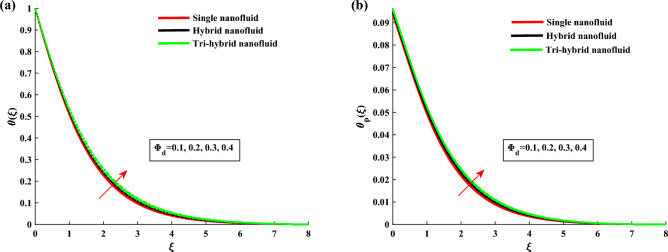
$\theta$ and $\theta _p$ variation against $\Phi _d$.

The non-dimensional ODE’s are solved by utilizing BVP4c technique. In Table 1 the thermo-physical characteristics of base fluid and nanosize particles are mentioned. For validation the present results are compared with existing literature, the results comparison is shown in Table 2. An excellent agreement is observed with the literature. The outcomes of this investigation are explained via Figs. 2, 3, 4, 5, 6, 7, 8, 9 and 10. Figure 2a,b depicts the fluctuation in $H_1$, $H_2$ w.r.t modified Hartmann number *C*. The excessing strength of *C* is due to the increment of outward electric field. In this scheme the wall parallel force (Lorentz force) restrain the boundary layer growth. Since the magnetic range decreases rapidly, therefore velocity profile increased. Physically the magnetic range generates the Lorentz force that’s in turn resisting the fluid flow. However in the present circumstance, the magnetic range decreases therefore the Lorentz force also decreases, as a result velocity profile increased. The magnitude of $H_2$ is decreases for higher values of *C*. It is ratified that the application of electro-magnetic field constructed as a Riga plate setting comfort to stable the rotatable flow. Figure 3a,b shows the impact of rotation parameter $\beta$ on Primary velocity $H_1$ and secondary velocity $H_2$. It is noted that with amplifying values of $\beta$ there is retardation in $H_1$. In case of $\beta =0$ (pure stretchable case) the velocity attains its highest values. Due to Coriolis forces, the fluid motion slows down. For higher values of $\beta$ the secondary velocity $H_2$ has the inverse behavior.

**Figure 11:**
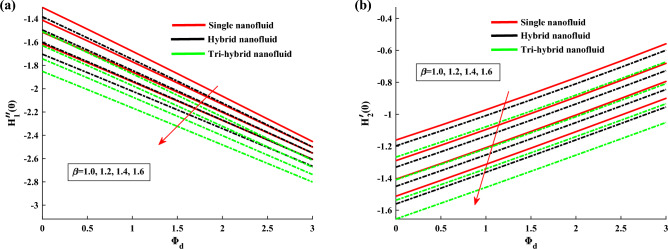
Skin friction variation against $\beta$ and $\phi _d$.

**Figure 12:**
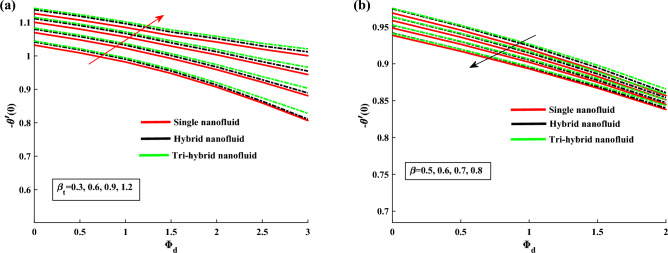
Nusselt number variation against $\beta _t$, $\beta$ and $\phi _d$.

Figure 4a,b demonstrate the influence of $\beta$ on the dusty phase fluid velocities. Here, $H_{1p}$ and $H_{2p}$ denote the MBL (momentum boundary layer) for dusty case in *x*-axis and *y*-axis. In dusty case of fluid the axial velocity decrease due to rising strength of rotation parameter and transverse velocity shows the opposite behavior against this parameter. Figure 5a,b indicates the fluid velocities for $\beta _v$. It reveals that the axial velocity of ternary fluid phase is depressed with higher input of $\beta _v$. Physically an increasing the dust particles mass concentration the dust particles weight is increased which decreases the fluid velocity. On the other hand transvers velocity shows the opposite behavior for increasing trend of $\beta _v$. Figure 6a,b demonstrate the effect of $\beta _v$ on the dusty phase fluid velocities. In dusty case of fluid the axial velocity increase due to rising values of dust particles mass concentration and against this parameter transverse velocity shows the opposite behavior. Figure 7a,b portrays the influences of dusty volume fraction variation on axial and transverse velocities. It is observed that by increasing the concentration of dust particles, the liquid becomes thick and creates more resistance, therefore axial velocity decreased. Due to rotation an opposite trend is noticed in transverse velocity. Figure 8a,b illustrates the impact of rotation parameter $\beta$ on fluid temperature and dusty phase of ternary fluid. It is observed that in dusty and ternary fluid phase, temperature increased with higher values of $\beta$. Basically, the energy development is satisfied on the base of a diffusion procedure because of increased rotation. Figure 9a,b show the thermal dusty parameter influence on $\theta$ and $\theta _p$. For amplifying values of $\beta _t$ the fluid flow is slow down therefore temperature is decreased. On the other hand higher values of $\beta _t$, in suspended debris enhance the friction force. Therefore dusty fluid temperature is increased. Figure 10a,b demonstrate the dusty volume fraction impact on temperature. For higher inputs, fluid temperature and dusty fluid temperature increases. Basically by increasing the dusty volume fraction thermal conductivity increased therefore temperature boost up. Figure 11a,b reveals the skin friction coefficient for distinct values of rotating parameter and dust particles concentration. It is noted that both primary and secondary velocities shows decreasing trend for higher input of rotating parameter. For increasing values of dust concentration the primary velocity decreases and secondary velocity shows the opposite behaviour. Figure 12a,b portray the Nusselt number against thermal dust factor, rotating parameter and dust particles concentration. Nusselt number shows the decreasing trend for higher values of dust particles concentration.

**Table 2:** Comparing the present numerical Nusselt number for *Pr* when all other parameters are zeros.

*Pr*	Ref.^39^	Ref.^40^	Present results
1.0	1.0000	1.0000	1.0000
3.0	1.92375	1.9238	1.9236
10.0	3.72061	3.7210	3.7206

## Conclusions

Numerical technique is conducted for dusty trihybrid Ellis nanofluid time-independent rotational flow over a stretching Riga plate The axial and transverse fluid velocities, nano size particles volume fraction and fluid temperature are evaluated for appropriate fluctuating inputs of sundries parameters (Supplementary Information). The main findings are mentioned briefly:

- For maximum inputs of $\beta$ and $\beta _v$, $H'_1$ and magnitude of $H_2$ decreases conspicuously.
- For higher values of modified magnetic parameter *C*, magnitude of $H_2$ and $H'_1$ enhance.
- The higher values of $\phi _d$, axial velocity decrease and transverse velocity show the opposite trend.
- In dusty phase the axial velocity increase and transverse velocity decrease against $\beta _v$ and $\beta$ shows the opposite trend.
- The enhancement in rotation and dust concentration parameter, temperature in trihybrid fluid and also in dusty phase increases.
- Fluid temperature profile increases against $\beta _t$ and show opposite behavior in dusty phase.
- It is also noted that ternary hybrid nanoliquid attains maximum temperature as compared to single and hybrid.
- In skin friction coefficient $\phi _d$ shows decreasing behaviour in axial velocity and opposite trend is noted against secondary velocity.
- Nusselt number increased against $\beta _t$ and opposite trend is investigated against $\beta$.
  Through this successful computational attempt, we have successfully expound the parameter effects on the dusty trihybrid Ellis fluid. This article may be extended for Oldroyd-B dusty nanoliquid, Maxwell dusty annaoliquid and Jffrey’s dusty nanoliquid.

### Supplementary Information

Supplementary Information.

## Data Availability

The data that support the findings of this study are available from the corresponding author upon reasonable request.

## List of symbols

*x*, *y*, *z*: Cartesian coordinates
$$\Omega $$: Constant velocity
$\rho _p$: Dust particles density
$C_p$: Dust particles concentration
*T*: Fluid temperature
$T_p$: Dust particles temperature
$c_p$: Specific thermal capacity of liquid
*C*: modified Hartman number
*Pr*: Prandtl factor
$$\beta $$: Rotation parameter
$\beta _t$: Thermal dust factor
*d*: Non-dimensional parameter
$\gamma _t$: Specified thermal factor
$\rho _p$: Dust particles density
*K*: Stoke’s drag constant
*L*: Micro-rotation factor
*C*: Modified Hartman parameter
*Pr*: Prandtl number
$\phi _d$: Concentration of dust particles
$$B_1$$: Fluid parameter
$\beta _v$: Velocity of fluid particles
$\gamma _v$: Dust particles mass concentration
$k_{Thnf}$: Thermal conductivity of trihybrid
