# Author Correction: Numerical investigation of dusty tri-hybrid Ellis rotating nanofluid flow and thermal transportation over a stretchable Riga plate

**DOI:** 10.1038/s41598-023-43647-0

**Published:** 2023-09-29

**Authors:** Humaira Sharif, Bagh Ali, Imran Siddique, Iqra Saman, Mohammed M. M. Jaradat, Mohammad Sallah

**Affiliations:** 1 Department of Mathematics, Government College University Faisalabad, Layyah Campus, Layyah, 31200 Pakistan; 2 School of Mechanical Engineering and Automation, Harbin Institute of Technology, Shenzhen, 518055 China; 3 Department of Mathematics, University of Management and Technology, Lahore, 54770 Pakistan; 4 Mathematics Program, Department of Mathematics, Statistics and Physics, College of Arts and Sciences, Qatar University, 2713 Doha, Qatar; 5 Applied Mathematical Physics Research Group, Physics Department, Faculty of Science, Mansoura University, Mansoura, 35516 Egypt; 6 Higher Institute of Engineering and Technology, New Damietta, Egypt

Correction to: *Scientific Reports* https://doi.org/10.1038/s41598-023-41141-1, published online 31 August 2023

The original version of this Article contained an error in the spelling of the author Mohammed Sallah which was incorrectly given as Muhammed Sallah.

The original Article has been corrected.

