# Supplementary material for: Numerical investigation of dusty tri-hybrid Ellis rotating nanofluid flow and thermal transportation over a stretchable Riga plate
Source: Sci Rep. 2023 Aug 31;13:14272. doi: 10.1038/s41598-023-41141-1 (PMC10471745; doi:10.1038/s41598-023-41141-1)
Supplement: Supplementary file 1 — Supplementary Information. [file 41598_2023_41141_MOESM1_ESM.zip › P22- dusty Ellis tri/huma paper.pdf]

Numerical investigation of dusty tri-hybrid Ellis rotating nanofluid flow and thermal transportation over a stretchable Riga plate[☆]

Humaira Sharif[a], Bagh Ali[a,b], Iqra Saman[c], Imran Siddique[c,*]

[a]Department of Mathematics, Government College University Faisalabad, Layyah Campus, Layyah 31200, Pakistan
[b]School of Mechanical Engineering and Automation, Harbin Institute of Technology, Shenzhen 518055, China
[c]Department of Mathematics, University of Management and Technology, Lahore 54770, Pakistan

Abstract

Due to high-ultra thermic significances, the nanosize materials are used in various chemical and mechanical engineering, modern technology and thermic engineering eras. For industrial growth of a country, one of the biggest challenges for engineers and scientists is improvement in thermal production and resources. In this study we analyzed the momentum and thermic aspects of MHD Ellis ternary nano material embedded with dust particles via stretchable Riga plate including volume concentration of dust material. The flow generating PDE's for two phase models are minimized into dimensionless nonlinear ODE's by using the right modification. To acquire the graphical results the BVP4c method was adopted in MATLAB software. Fundamental aspects affecting velocity and temperature have investigated through graphs. Additionally Nusselt number and skin friction have also been evaluated. Compared it with previous literature to check the validity of results. Finding reveals that as compared to dusty phase the performance of trihybrid nano phase thermal transport is improved. Moreover, the temperature profile increases for rotational and volume fraction dust particles parameter. Dusty fluids are used in numerous manufacturing and engineering sectors, like petroleum transport, car smoke emissions, caustic granules in mining and power plant pipes.

Keywords: Ellis fluid, volume fraction, rotating surface, dust particles, tri-hybrid nanofluid, Riga plate, numerical solution
2010 MSC: 00-01, 99-00

1. Introduction

In heat transport system the application of nanomaterial play a fundamental role in different industrial procedures involving thermic and chemical operations. In numerous heat transport system, distinct liquids have been used as a thermal porters. Heat transport fluids are valuable to a various applications like automobile system [1, 2], heat transfer in power plants [3, 4] and system of temperature changing [5]. In heat transfer fluids the thermal conductivity plays a significant role on the performance of heat transport procedures and device performance. Heat transposition may be accomplished by using nanoliquids. Sharif et al. [6] analyzed the energy effects on Eyring's nanofluid with microorganisms. Hussain et al. [7] investigated the Brownian motion impact in the presence of motile microorganisms.

[☆]Fully documented templates are available in the elsarticle package on CTAN.
[*]Corresponding author: imransmsrazi@gmail.com

Nanofluids are produced by mixing micro size particles in base liquid like water, minerals, air etc. Although when more than one kind of nano materials are extant in base liquid, the nanoliquids is transferred into hybrid nanoliquids. Hybrid nanoliquids demonstrate an exceptional performance as compared to mono nanoliquids [8]. Therefore hybrid nanoliquids are widely used to enhance the better heat transport [9]. Timofeeva et al.[10] demonstrated that the dynamic viscosity of alumina-based nanofluids varies with the geometry of the nanoparticles at different temperatures. Surface charge is linked to these variations in the agglomeration and interactions between each form of nanoparticle (platelets, bricks, blades, and cylinders) and the base fluid. This is in strong agreement with Sahu and Sarkar's conclusion [11], which states that Nanoparticle morphologies affect both the exergetic and energetic performance. Jiang et al.[12] described the dynamics of nanofluids resulting from thermo-capillary convection created by various five nanoparticle forms (sphere, blade, brick, cylinder, and platelet). The amount of thermo-capillary convection was found to be at its highest in a nanofluid made of spherical nanoparticles, and at its lowest in platelet-shaped nanoparticles. Additionally, blade nanoparticles had a 22.8% Nusselt number increase, compared to a 2.8% rise in blade-shaped nanoparticles. Algehyne et al. [13] reported numerically trihybrid nanoliquids flow using the concept of non-Fourier's and diffusion factor. They revealed that as compared to single nanoliquids, hybrid and ternary nanoliquids have outstanding tendency for liquid energy and rate of velocity propagation. More studies on nano fluid flow subject to various geometries are cited in [14, 15, 16, 17].

In modern age, dusty fluid flow model has unique investigators interest because of its two phase system. This impact appears in liquids flow with the distribution of solid particles. For instance, the reaction of chemical through which droplets are generated with the dusty air velocity and consolidation of dusty particles in difficulties of fluidization. The significant former for planetary structure is constructed by mixing dust and gas particles known as cosmic dust. Many researchers utilized the model of dusty phase with boundary conditions and various flow structures. Therefore, the outcomes they provided are numerical and approximate approaches.First of all Saffman [18] gave the idea of dusty fluid. By utilizing the theory of Stoke's drag he derived the equations for dusty liquid. He also observed that heat transport rate increased by using suspending dusty particles. Ezzat et al.[19] analyzed the dusty liquid transfer with free convection heat transport on a planar surface in the existing of porous media. Sivaraj and Kumar [20] investigated the MHD unsteady dusty fluid along an irregular surface with variation of mass diffusion. Dey and Chutia [21] presented the dusty nanoliquids flow with bio-convection past a vertically stretchable surface. Rehman et al. [22] examined the dusty Casson nanofluid past a stretchable surface with magnetic field and Darcy forchheimer law. They observed that for higher values of time relaxation the energy profile decreases in both phases.

Knowledge about the rotatable fluid flow is very useful for mechanical engineering, radiators, chemical industry, bio-medical spin coating, centrifugal etc. They are utilized for rotatable machinery, devices of computer storage, lubrications and in various engineering field. Hussain et al. [23] conducted research to overcome the unstable nanofluid magnetohydrodynamic flow through the permeable channel past the rotating device's moving surface while accounting for mass and heat transfer. Khan et al. [24] looked into the conformational entropy of bio-convection nanofluid flow between two stretchy rotating disks. Nazar et al. [25] promoted flow difficulties with instability. Their findings indicate a smooth transition from the initial unsteady flow to the final smooth flow. Ali et al. [26] deliberated the

unsteady rotatable flux of a Maxwell fluid past a stretchable cylinder. Hussain et al.[27] studied the Darcy -forchheimer nanoliquids flow past a rotatable disk. Liu et al.[28] inquired the rotatable flux dynamics in frictional stir welding. More investigations on rotatable fluid flow subject to various geometries are cited in [29, 30, 31, 32].

Various applications of trihybrid dusty fluid in present technology, developed a motivation to formulate this article. The non-Newtonian dusty fluids have widespread applications in many engineering field and industries, like production of cement, nuclear reactors, thermal exchanger, petroleum extraction, pipe industry, metalworking, etc. By analyzing the aloft mentioned literature, we conclude that analysis on the two phase dusty trihybrid Ellis fluid through a rotatable Riga plate did not performed. The nonlinear problem is sort out via numerically by using BVP4c approach. The including parameters are drawn graphically, to investigate the fluctuation of various profiles. To investigate the variations in physical quantities the present outcomes have been compared with existing literature.

2. Mathematical-Formulation

We assume steady, 3D rotatable of dusty tri-hybrid Ellis nanofluid flow by a stretching Riga plate. The combination of magnets and electrodes the Riga plate constructed. Due to electro-magnetic field of a Riga plate, force that is parallel to plate is Lorentz force. The plate ia stretchable in xy direction and liquid placed with the z direction. Along the z-direction the fluid rotate with Ω angular velocity. The velocity of primary flow is positive, the second body force is negative, that establish a negative effect on secondary velocity of fluid. Dust particles and fluid were assumed to be stable. The fluid is incompressible , therefore the dust particles density is constant and between the dust particles energy is prevent. Volume fraction of dusty particles has also been into account. Further, The plate having stretched velocity U_w along x-axis. Due to tri-hybrid nanofluid is considered a stable mixture, therefore nano size particles agglomeration is ignored. zero velocity is assumed at ambient surafce. T_w and T_∞ are the wall and ambient temperature. The model is sketched in Fig.1.

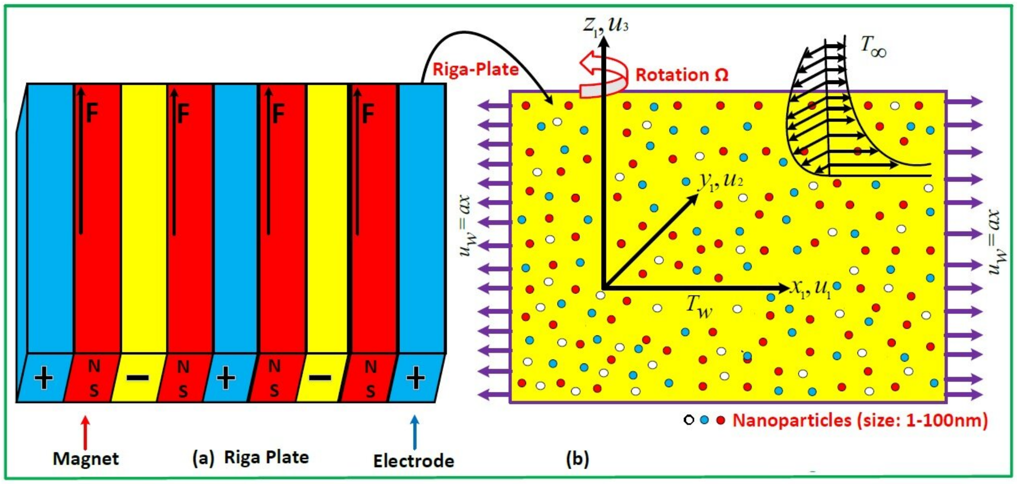

Figure 1: Flow model

Considering the aloft conditions, the conservation of momentum and temperature equations can be mentioned as [33, 34]:

$$\frac{\partial u_1}{\partial x}+\frac{\partial u_2}{\partial y}+\frac{\partial u_3}{\partial z}=0, \tag{1}$$

$$(1-\Phi_d)\rho_{Thnf}(u_1\frac{\partial u_1}{\partial x}+u_2\frac{\partial u_1}{\partial y}+u_3\frac{\partial u_1}{\partial z}-2\Omega u_2)=(1-\Phi_d)\mu_{Thnf}\frac{\partial^2 u_1}{\partial z^2}+$$

$$(1-\Phi_d)\mu_{Thnf}\frac{\partial}{\partial z}\left[\frac{1}{1+\left(\frac{\partial u_1}{\tau_0^2\partial z}\right)^{\alpha-1}}\frac{\partial u_1}{\partial z}\right]+KL(u_1p-u_1)+\frac{\pi j_0 M_0}{8}e^{-(\pi/a)z}, \tag{2}$$

$$(1-\Phi_d)\rho_{Thnf}(u_1\frac{\partial u_2}{\partial x}+u_2\frac{\partial u_2}{\partial y}+u_3\frac{\partial u_2}{\partial z}+2\Omega u_1)=(1-\Phi_d)\mu_{Thnf}\frac{\partial^2 u_2}{\partial z^2}+$$

$$(1-\Phi_d)\mu_{Thnf}\frac{\partial}{\partial z}\left[\frac{1}{1+\left(\frac{\partial u_2}{\tau_0^2\partial z}\right)^{\alpha-1}}\frac{\partial u_2}{\partial z}\right]+KL(u_2p-u_2), \tag{3}$$

$$(\rho C_P)_{Thnf}(u_1\frac{\partial T}{\partial x}+u_2\frac{\partial T}{\partial y}+u_3\frac{\partial T}{\partial z})=k_{Thnf}\frac{\partial^2 T}{\partial z^2}+\frac{\rho_p C_p}{\tau_T}(T_p-T). \tag{4}$$

For dusty phase

$$\frac{\partial u_{1p}}{\partial x}+\frac{\partial u_{2p}}{\partial y}+\frac{\partial u_{3p}}{\partial z}=0, \tag{5}$$

$$(u_{1p}\frac{\partial u_{1p}}{\partial x}+u_{2p}\frac{\partial u_{1p}}{\partial y}+u_{3p}\frac{\partial u_{1p}}{\partial z}-2\Omega u_{2p})=\frac{KL}{\rho}(u_1-u_{1p}), \tag{6}$$

$$(u_{1p}\frac{\partial u_{2p}}{\partial x}+u_{2p}\frac{\partial u_{2p}}{\partial y}+u_{3p}\frac{\partial u_{2p}}{\partial z}+2\Omega u_{1p})=\frac{KL}{\rho}(u_2-u_{2p}), \tag{7}$$

$$\rho_p c_p(u_{1p}\frac{\partial T_p}{\partial x}+u_{2p}\frac{\partial T_p}{\partial y}+u_{3p}\frac{\partial T_p}{\partial z})=\frac{\rho_p c_p}{\tau_T}(T-T_p). \tag{8}$$

The appropriate boundary conditions are [35, 36]:

$$u_1=U_w=ax,\ u_2=u_3=0,\ T=T_w,\ as\ z=0, \tag{9}$$

$$u_1p=u_1=0,\ u_2p=u_2=0,\ u_3p\to u_3,\ T\to T_\infty,\ T_p\to T_\infty,\ as\ z\to\infty. \tag{10}$$

Here, (u_1, u_2, u_3) are velocities component in (x, y, z) directions. Dust particles velocity components are represented by (u_1p, u_2p, u_3p), Ω denotes the constant velocity, U_w denotes the stretchable velocity component in x-direction, a is stretchable constant rate (a is positive), ρ_{Thnf} is density tri-hybrid nanoliquid, ρ_p is dust particles density, C_p is dust particles concentration, T is liquid temperature, T_p is dust particles temperature, c_p is specific thermal capacity of liquid, k_{Thnf} is thermic conductivity of tri-hybrid, τ_T is thermic stability time, K is constant of Stoke's drag and L is micro-rotation factor.

3. Rheological and thermophysical characteristics

The thermophysical characteristics of TiO_2, MgO, $COFe_2O_4$ ternary hybrid nanoliquid are [37]:

1. Viscosity

$$\mu_{Thnf} = \mu_f(1-\Phi_1)^{-2.5}(1-\Phi_2)^{-2.5}(1-\Phi_3)^{-2.5},$$

2. Density

$$\rho_{Thnf} = \left[(1-\Phi_1)\left[(1-\Phi_2)\bigg((1-\Phi_3)(\rho_f+\rho_3\Phi_3)\bigg)+(\rho_2\Phi_2)\right]+\rho_1\Phi_1\right],$$

3. Heat Capcity

$$(\rho C_p)_{Thnf} = (1-\Phi_1)\bigg((1-\Phi_2)\left[(1-\Phi_3)(\rho C_p)_f+(\rho C_p)_{s_3}\Phi_3\right]+(\rho C_p)_{s_2}\Phi_2\bigg)+(\rho C_p)_{s_1}\Phi_1,$$

4. Thermal Conductivity

$$\frac{k_{nf}}{k_f} = \frac{k_3+2k_{nf}-2\Phi_3(k_f-k_3)}{k_3+2k_{nf}+\Phi_3(k_f-k_3)},$$
$$\frac{k_{hnf}}{k_{nf}} = \frac{k_2+2k_{nf}-2\Phi_2(k_{nf}-k_2)}{k_2+2k_{nf}+\Phi_2(k_{nf}-k_2)},$$
$$\frac{k_{Thnf}}{k_{hnf}} = \frac{k_1+2k_{nf}-2\Phi_1(k_{hnf}-k_1)}{k_1+2k_{nf}+\Phi_1(k_{hnf}-k_1)},$$

Table 1: Thermophysical properties of nano size particles and water base fluid .

Physical properties	TiO_2	MgO	$COFe_2O_4$	H_2O
ρ	4250.0	3560	4907	0997.1
C_p	686.2	955	700	4179
κ	8.9538	45	3.7	0.613

4. Similarity conversion

We assumed the following appropriate transformation [38].

$$\left.\begin{aligned} \xi = \sqrt{\frac{a}{\nu}}z,\ u_1 = axH_1{}'(\xi),\ u_2 = axH_2(\xi),\ u_3 = -(a\nu)^{\frac{1}{2}}H_1(\xi),\\ \theta(\eta)(T_w-T_\infty) = T-T_\infty,\\ \xi = \sqrt{\frac{a}{\nu}}z,\ u_{1p} = axH_{1p}{}'(\xi),\ u_{2p} = axH_{2p}(\xi),\ u_{3p} = -(a\nu)^{\frac{1}{2}}H_{1p}(\xi),\\ \theta_p(\eta)(T_w-T_\infty) = T_P-T_\infty. \end{aligned}\right\} \tag{11}$$

Eq. (1) is identically satisfied. By utilizing the aloft mentioned transformations in Eqs. (2)-(10), we get

$$\frac{(1-\Phi_d)}{Z_1Z_2}\left(\frac{1+(2-\alpha)(B_1'H_1'')^{\alpha-1}}{(1+(B_1'H_1'')^{\alpha-1})^2}\right)H_1''' + (1-\Phi_d)(H_1H_1''+2\beta H_2 - H_1'^2) + \frac{\beta_v\gamma_v}{Z_2}(H_{1p}'-H_1') + \frac{C}{Z_2}e^{-d\eta} = 0, \tag{12}$$

$$\frac{(1-\Phi_d)}{Z_1Z_2}\left(\frac{1+(2-\alpha)(B_1'H_1'')^{\alpha-1}}{(1+(B_1'H_1'')^{\alpha-1})^2}\right)H_2'' + (1-\Phi_d)(H_1H_2'-2\beta H_1' - H_1'H_2) + \frac{\beta_v\gamma_v}{Z_2}(H_{2p}-H_2) = 0, \tag{13}$$

$$\frac{1}{Pr}\frac{Z_4}{Z_5}\theta'' + H_1\theta' + \gamma_t\beta_t(\theta_p-\theta) = 0, \tag{14}$$

For the dusty case

$$H_{1p}'^2 - H_1pH_1''p - 2\beta H_2p + \beta_v\gamma_v(H_1'p - H_1') = 0, \tag{15}$$

$$H_{1p}'H_{2p} - H_{1p}H_{2p}' + 2\beta H_{1p}' + \beta_v\gamma_v(H_2p - H_2) = 0, \tag{16}$$

$$H_{1p}\theta_p' + \beta_t\gamma_t(\theta-\theta_p) = 0. \tag{17}$$

Boundary conditions are

$$\left.\begin{aligned} H_1(0)=0,\ H_1'(0)=1,\ H_2(0)=0,\ \theta(0)=1,\ at\,\xi=0,\\ H_1'(\infty)\to 0,\ H_2(\infty)\to 0,\ H_{1p}'(\infty)\to 0,\ H_{2p}(\infty)\to 0,\ \theta(\infty)\to 0,\\ \theta_p(\infty)\to 0,\ H_{1p}(\infty)\to H_1(\infty),\ at\,\xi\to\infty. \end{aligned}\right\} \tag{18}$$

Here, C is modified Hartman parameter, d is non-dimensional parameter, β is rotation parameter, ϕ_d is concentration of dust particles, Pr is Prandtl factor, B_1 is fluid parameter, β_t is thermal dust factor, γ_t is specified thermal ratio, β_v is velocity of fluid particles, γ_v is dust particles mass concentration, Mathematically,

,

$$\beta = \frac{\Omega}{a},\ Pr = \frac{(\mu c_p)_f}{k_f},\ B_1 = \frac{1}{\tau_0^2}\sqrt{\frac{a(ax)^2}{\nu_f}}, \alpha = \frac{\pi}{p}\sqrt{\frac{\nu_f}{a}},\ \beta_t = \frac{1}{a\tau_T}, \gamma_t = \frac{c_p}{c_m},\ C = \frac{\pi}{\rho_f 8a^2},$$
$$\beta_v = \frac{K}{am}, \gamma_v = \frac{Lm}{\rho},\ Z_1 = \frac{\mu_(thnf)}{\mu_f},\ Z_2 = \frac{\rho_(thnf)}{\rho_f},\ Z_4 = \frac{k_(thnf)}{k_f},\ Z_5 = \frac{(\rho C_p)_(thnf)}{(\rho C_p)_f},$$

The physical quantities are Nusselt number and skin friction coefficient are defined as:

$$\left.\begin{aligned} Cf_x &= \frac{\tau_(xz)}{U_w^2\rho_nf},\\ Cf_y &= \frac{\tau_(yz)}{U_w^2\rho_nf},\\ Nu &= \frac{xq_w}{(T_w-T_\infty)k_f}, \end{aligned}\right\} \tag{19}$$

Where Cf_x, Cf_y are skin friction coefficients along x and y-axis, Nu is Nusselt number. The non-dimensional form of Nusselt number and skin friction coefficient are as follows:

$$\begin{cases} Cf_x(Re_x)^{0.5} = 1/Z_1\left(\frac{H_1''(0)}{1+(B_1H_1''(0))^{\alpha-1}}\right), Cf_y(Re_x)^{0.5} = 1/Z_1\left(\frac{H_2'(0)}{1+(B_1H_2'(0))^{\alpha-1}}\right),\\ Nu_xRe_x^{-0.5} = -Z_4\theta'(0), \end{cases} \tag{20}$$

Where $Re_x = \frac{xU_w}{\nu_f}$ is the Reynolds number.

5. Solution method

The bvp4c technique is commonly used for solving the initial value problems. This technique is very stable and easy to implement.The non-linear equations (12)-(17) with boundary conditions (18) are solved numerically by using bvp4c method in MATLAB environment. In this method, the system of differential equations (12)-(17) is reduced to first order ODE's.

$$H_1(\xi) = y_1, H_1'(\xi) = y_2, H_1''(\xi) = y_3,$$

$$y_3' = \frac{-Z_1Z_2}{(1-\Phi_d)}\left(\frac{(1+(B_1'y_3)^{\alpha-1})^2}{1+(2-\alpha)(B_1'y_3)^{\alpha-1}}\right)\left[(1-\Phi_d)(y_1y_3+2\beta y_4-y_2^2)+\frac{\beta_v\gamma_v}{Z_2}(y_9-y_2)+\frac{C}{Z_2}e^{-d\eta}\right]=0,$$

$$H_2'(\xi) = y_5,$$

$$y_5' = \frac{-Z_1Z_2}{(1-\Phi_d)}\left(\frac{(1+(B_1'y_3)^{\alpha-1})^2}{1+(2-\alpha)(B_1'y_3)^{\alpha-1}}\right)\left[(1-\Phi_d)(y_1y_5-2\beta y_2-y_2y_4)+\frac{\beta_v\gamma_v}{Z_2}(y_10-y_4)\right]=0,$$

$$\theta'(\xi) = y_7,$$

$$y_7' = \frac{-PrZ_5}{Z_4}\left[y_1y_7+\gamma_t\beta_t(y_{11}-y_6)\right]=0,$$

$$H_{1p}' = y_9,$$

$$y_9' = 1/y_8\left[y_9^2-2\beta y_4+\beta_v\gamma_v(y_9-y_2)\right],$$

$$H_{2p} = y_10,$$

$$y_{10}' = 1/y_8\left[y_9y_{10}+2\beta y_9+\beta_v\gamma_v(y_{10}-y_4)\right],$$

$$\theta_p = y_{11},$$

$$y_{11}' = -1/y_8\left[\beta_t\gamma_t(y_6-y_{11})\right],$$

With the relevant conditions are:

$$y_1(0)=0,\ y_2(0)=1,\ y_4(0)=0,\ y_6(0)=1,$$

$$y_2(\infty)\to 0,\ y_4(\infty)\to 0,\ y_6(\infty)\to 0,\ y_9(\infty)=\to 0,\ y_{10}(\infty)\to 0,$$

$$y_{11}(\infty)\to 0,\ y_8(\infty)\to y_1(0).$$

6. Results and Discussion

The non-dimensional ODE's are solved by utilizing BVP4c technique. In table 1 the thermo-physical characteristics of base fluid and nanosize particles are mentioned. For validation the present results are compared with existing literature, the results comparison is shown in Table 2. An excellent agreement is observed with the literature. The outcomes of this investigation are explained via Fig. 2 to Fig. 10. Fig 2 (a-b) depicts the fluctuation in H_1, H_2 w.r.t

modified Hartmann number C. The excessing strength of C is due to the increment of outward electric field. In this scheme the wall parallel force (Lorentz force) restrain the boundary layer growth. Since the magnetic range decreases rapidly, therefore velocity profile increased. Physically the magnetic range generates the Lorentz force that's in turn resisting the fluid flow. However in the present circumstance, the magnetic range decreases therefore the Lorentz force also decreases, as a result velocity profile increased. The magnitude of H_2 is decreases for higher values of C. It is ratified that the application of electro-magnetic field constructed as a Riga plate setting comfort to stable the rotatable flow. Fig 3 (a-b) shows the impact of rotation parameter β on Primary velocity H_1 and secondary velocity H_2. It is noted that with amplifying values of β there is retardation in H_1. In case of $\beta = 0$ (pure stretchable case) the velocity attains its highest values. Due to Coriolis forces, the fluid motion slows down. For higher values of β the secondary velocity H_2 has the inverse behavior.

Fig 4 (a-b) demonstrate the influence of β on the dusty phase fluid velocities. Here, H_{1p} and H_{2p} denote the MBL (momentum boundary layer) for dusty case in x-axis and y-axis. In dusty case of fluid the axial velocity decrease due to rising strength of rotation parameter and transverse velocity shows the opposite behavior against this parameter. Fig 5 (a-b) indicates the fluid velocities for β_v. It reveals that the axial velocity of ternary fluid phase is depressed with higher input of β_v. Physically an increasing the dust particles mass concentration the dust particles weight is increased which decreases the fluid velocity. On the other hand transvers velocity shows the opposite behavior for increasing trend of β_v. Fig 6 (a-b) demonstrate the effect of β_v on the dusty phase fluid velocities. In dusty case of fluid the axial velocity increase due to rising values of dust particles mass concentration and against this parameter transverse velocity shows the opposite behavior. Fig 7 (a-b) portrays the influences of dusty volume fraction variation on axial and transverse velocities. It is observed that by increasing the concentration of dust particles, the liquid becomes thick and creates more resistance, therefore axial velocity decreased. Due to rotation an opposite trend is noticed in transverse velocity. Fig 8 (a-b) illustrates the impact of rotation parameter β on fluid temperature and dusty phase of ternary fluid. It is observed that in dusty and ternary fluid phase, temperature increased with higher values of β. Basically, the energy development is satisfied on the base of a diffusion procedure because of increased rotation. Fig 9 (a-b) show the thermal dusty parameter influence on θ and θ_p. For amplifying values of β_t the fluid flow is slow down therefore temperature is decreased. On the other hand higher values of β_t, in suspended debris enhance the friction force. Therefore dusty fluid temperature is increased. Fig 10 (a-b) demonstrate the dusty volume fraction impact on temperature. For higher inputs, fluid temperature and dusty fluid temperature increases. Basically by increasing the dusty volume fraction thermal conductivity increased therefore temperature boost up.Fig 11 (a-b) reveals the skin friction coefficient for distinct values of rotating parameter and dust particles concentration. It is noted that both primary and secondary velocities shows decreasing trend for higher input of rotating parameter. For increasing values of dust concentration the primary velocity decreases and secondary velocity shows the opposite behaviour. Fig 12 (a-b) portray the Nusselt number against thermal dust factor, rotating parameter and dust particles concentration. Nusselt number shows the decreasing trend for higher values of dust particles concentration.

Table 2: Comparing the present numerical Nusselt number for Pr when all other parameters are zeros.

Pr	Ref. [39]	Ref. [40]	Present results
1.0	1.0000	1.0000	1.0000
3.0	1.92375	1.9238	1.9236
10.0	3.72061	3.7210	3.7206

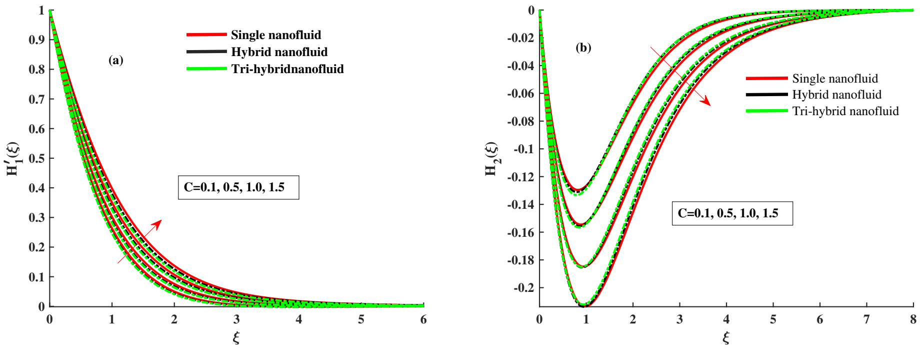

Figure 2: H_1' **and** H_2 **variation against** C**.**

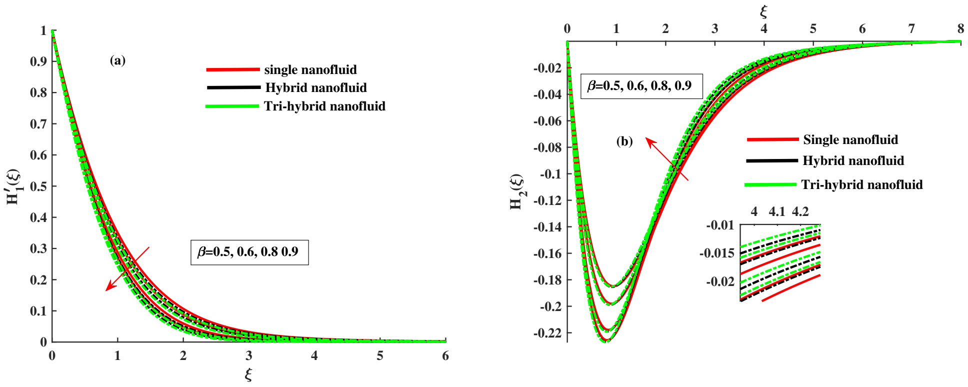

Figure 3: H_1' **and** H_2 **variation against** β**.**

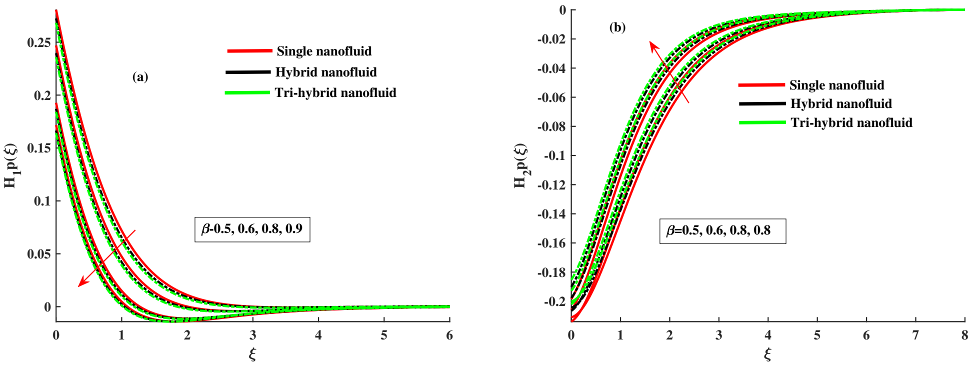

Figure 4: H_{1p} **and** H_{2p} **variation against** β**.**

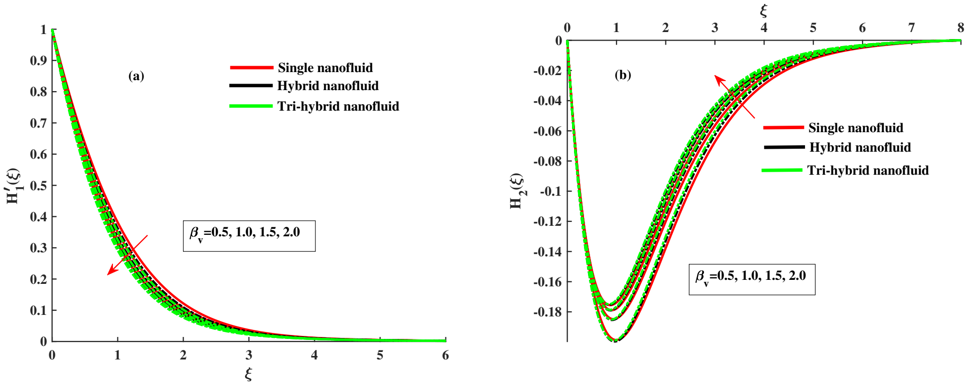

Figure 5: H_1' **and** H_2 **variation against** β_v**.**

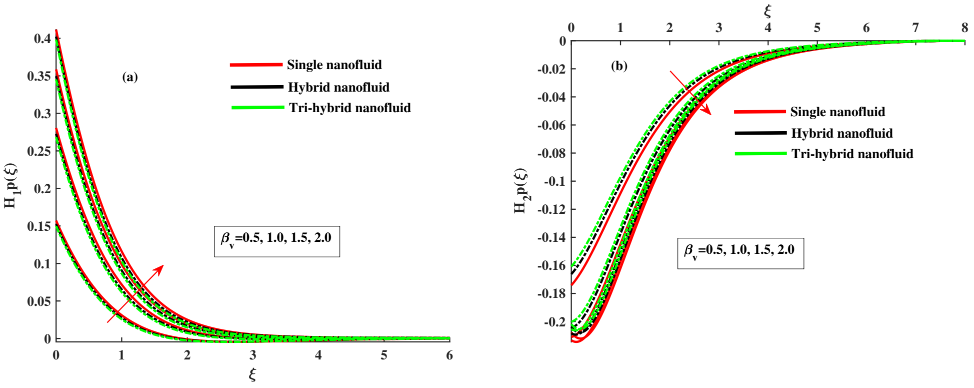

Figure 6: H_{1p} **and** H_{2p} **variation against** β_v**.**

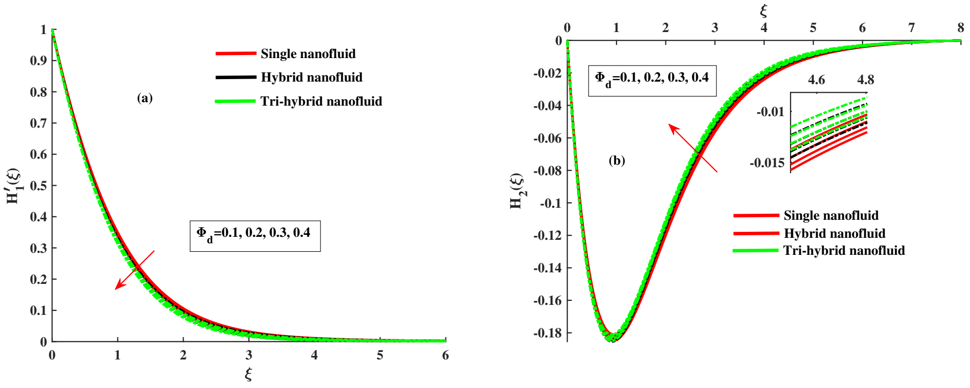

Figure 7: H_1' **and** H_2 **variation against** ϕ_d**.**

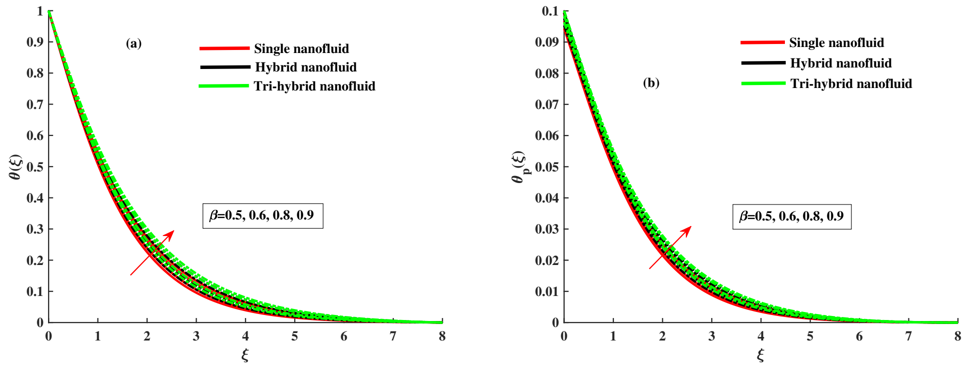

Figure 8: θ **and** θ_p **variation against** β**.**

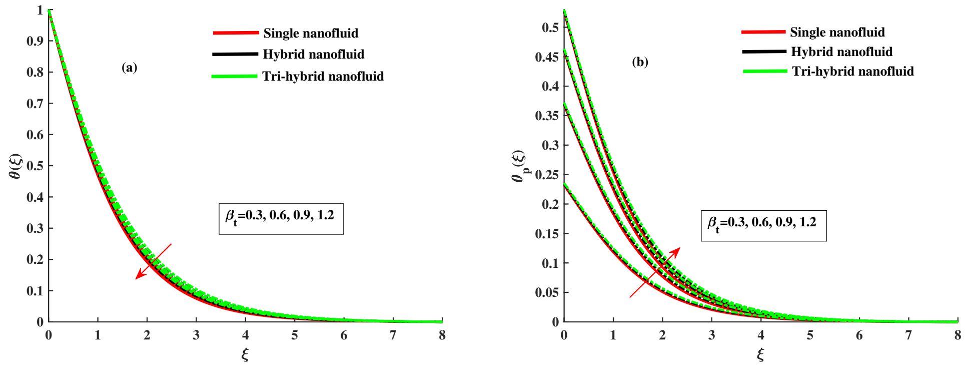

Figure 9: θ **and** θ_p **variation against** β_t**.**

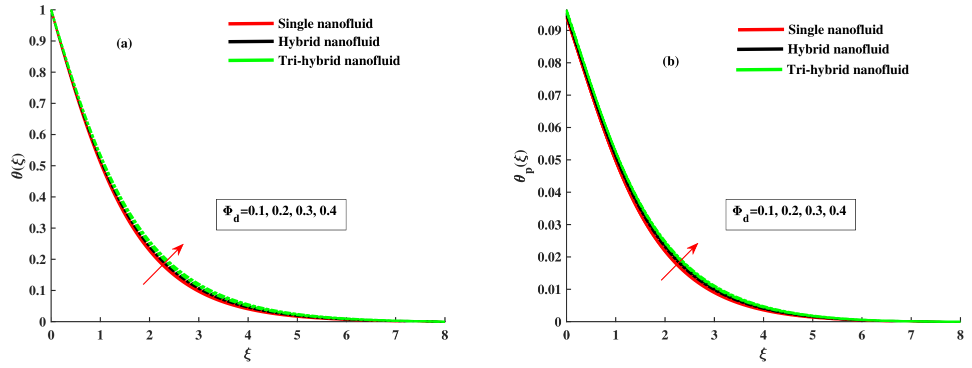

Figure 10: θ **and** θ_p **variation against** Φ_d**.**

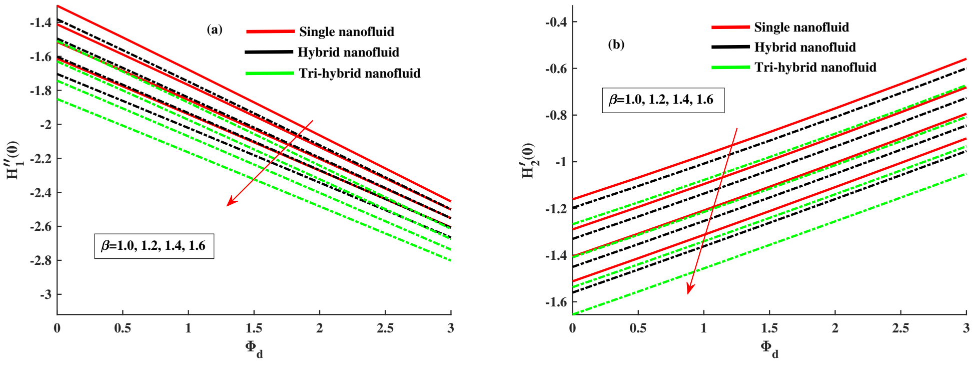

Figure 11: Skin friction variation against β **and** ϕ_d**.**

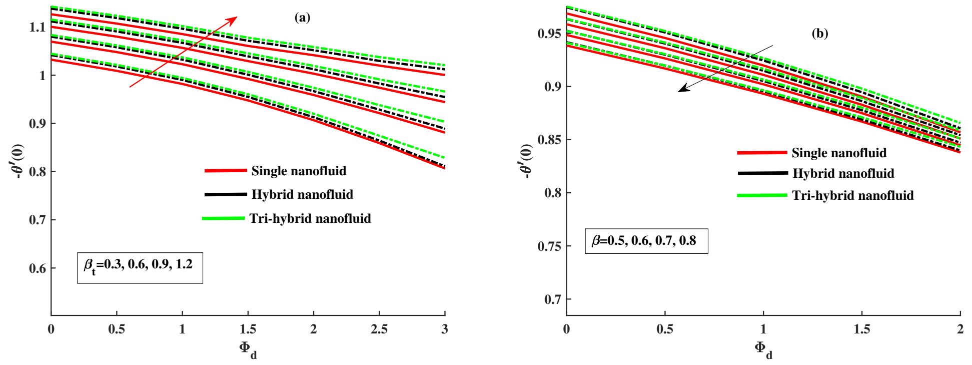

Figure 12: Nusselt number variation against β_t**,** β **and** ϕ_d**.**

7. Conclusions

Numerical technique is conducted for dusty trihybrid Ellis nanofluid time-independent rotational flow over a stretching Riga plate The axial and transverse fluid velocities, nano size particles volume fraction and fluid temperature are evaluated for appropriate fluctuating inputs of sundries parameters. The main findings are mentioned briefly:

- For maximum inputs of β and β_v, H_1' and magnitude of H_2 decreases conspicuously.
- For higher values of modified magnetic parameter C, magnitude of H_2 and H_1' enhance.
- The higher values of ϕ_d, axial velocity decrease and transverse velocity show the opposite trend.
- In dusty phase the axial velocity increase and transverse velocity decrease against β_v and β shows the opposite trend.
- The enhancement in rotation and dust concentration parameter, temperature in trihybrid fluid and also in dusty phase increases.
- Fluid temperature profile increases against β_t and show opposite behavior in dusty phase.
- It is also noted that ternary hybrid nanoliquid attains maximum temperature as compared to single and hybrid.
- In skin friction coefficient ϕ_d shows decreasing behaviour in axial velocity and opposite trend is noted against secondary velocity.
- Nusselt number increased against β_t and opposite trend is investigated against β.

  Through this successful computational attempt, we have successfully expound the parameter effects on the dusty trihybrid Ellis fluid. This article may be extended for Oldroyd-B dusty nanoliquid, Maxwell dusty annaoliquid and Jffrey's dusty nanoliquid.

8. Statement of data availability

The data that support the findings of this study are available from the corresponding author upon reasonable request.

9. Nomenclature

x, y, z	Cartesian coordinates
Ω	Constant velocity
ρ_p	Dust particles density
C_p	Dust particles concentration
T	Fluid temperature
T_p	Dust particles temperature
c_p	Specific thermal capacity of liquid
C	modified Hartman number
Pr	Prandtl factor
β	Rotation parameter
β_t	Thermal dust factor
d	Non-dimensional parameter
γ_t	Specified thermal factor
ρ_p	Dust particles density
K	Stoke's drag constant
L	Micro-rotation factor
C	Modified Hartman parameter
Pr	Prandtl number
ϕ_d	Concentration of dust particles
B_1	Fluid parameter
β_v	Velocity of fluid particles
γ_v	Dust particles mass concentration
k_{Thnf}	Thermal conductivity of trihybrid

[1] S. U. Choi, J. A. Eastman, Enhancing thermal conductivity of fluids with nanoparticles, Tech. rep., Argonne National Lab., IL (United States) (1995).

[2] Y. Xuan, Q. Li, Heat transfer enhancement of nanofluids, International Journal of heat and fluid flow 21 (1) (2000) 58–64.

[3] A. G. Memon, R. A. Memon, Thermodynamic analysis of a trigeneration system proposed for residential application, Energy Conversion and Management 145 (2017) 182–203.

[4] L. Coco-Enríquez, J. Muñoz-Antón, J. Martínez-Val, New text comparison between co2 and other supercritical working fluids (ethane, xe, ch4 and n2) in line-focusing solar power plants coupled to supercritical brayton power cycles, International Journal of Hydrogen Energy 42 (28) (2017) 17611–17631.

[5] M. Hashemian, S. Jafarmadar, J. Nasiri, H. S. Dizaji, Enhancement of heat transfer rate with structural modifi-

cation of double pipe heat exchanger by changing cylindrical form of tubes into conical form, Applied Thermal Engineering 118 (2017) 408–417.

[6] H. Sharif, M. N. Naeem, M. A. Khadimallah, H. Ayed, S. M. Bouzgarrou, A. F. Al Naim, S. Hussain, M. Hussain, Z. Iqbal, A. Tounsi, Energy effects on mhd flow of eyring's nanofluid containing motile microorganism, Advances in concrete construction 10 (4) (2020) 357–367.

[7] M. Hussain, H. Sharif, M. A. Khadimallah, H. Ayed, E. M. Banoqitah, H. Loukil, I. Ali, S. Mahmoud, A. Tounsi, Numerical calculations for bioconvection mhd casson nanofluid flow: Study of brownian motion, COMPUTERS AND CONCRETE 30 (2) (2022) 143–150.

[8] J. Sarkar, P. Ghosh, A. Adil, A review on hybrid nanofluids: recent research, development and applications, Renewable and Sustainable Energy Reviews 43 (2015) 164–177.

[9] U. Nazir, M. Nawaz, S. O. Alharbi, Thermal performance of magnetohydrodynamic complex fluid using nano and hybrid nanoparticles, Physica A: Statistical Mechanics and its Applications 553 (2020) 124345.

[10] E. V. Timofeeva, J. L. Routbort, D. Singh, Particle shape effects on thermophysical properties of alumina nanofluids, Journal of applied physics 106 (1) (2009) 014304.

[11] M. Sahu, J. Sarkar, Steady-state energetic and exergetic performances of single-phase natural circulation loop with hybrid nanofluids, Journal of Heat Transfer 141 (8).

[12] Y. Jiang, X. Zhou, Y. Wang, Effects of nanoparticle shapes on heat and mass transfer of nanofluid thermocapillary convection around a gas bubble, Microgravity Science and Technology 32 (2020) 167–177.

[13] E. A. Algehyne, H. F. Alrihieli, M. Bilal, A. Saeed, W. Weera, Numerical approach toward ternary hybrid nanofluid flow using variable diffusion and non-fourier's concept, ACS omega 7 (33) (2022) 29380–29390.

[14] S. M. Hussain, Numerical assessment of a sutterby hybrid nanofluid over a stretching sheet with a particle shape factor, Waves in Random and Complex Media (2023) 1–17.

[15] S. M. Hussain, Dynamics of radiative williamson hybrid nanofluid with entropy generation: significance in solar aircraft, Scientific Reports 12 (1) (2022) 8916.

[16] B. Ali, T. Thumma, D. Habib, S. Riaz, et al., Finite element analysis on transient mhd 3d rotating flow of maxwell and tangent hyperbolic nanofluid past a bidirectional stretching sheet with cattaneo christov heat flux model, Thermal Science and Engineering Progress 28 (2022) 101089.

[17] N. Acharya, S. Maity, P. K. Kundu, Entropy generation optimization of unsteady radiative hybrid nanofluid flow over a slippery spinning disk, Proceedings of the Institution of Mechanical Engineers, Part C: Journal of Mechanical Engineering Science 236 (11) (2022) 6007–6024.

[18] P. Saffman, On the stability of laminar flow of a dusty gas, Journal of fluid mechanics 13 (1) (1962) 120–128.

[19] M. A. Ezzat, A. El-Bary, M. Morsey, Space approach to the hydro-magnetic flow of a dusty fluid through a porous medium, Computers & Mathematics with Applications 59 (8) (2010) 2868–2879.

[20] R. Sivaraj, B. R. Kumar, Unsteady mhd dusty viscoelastic fluid couette flow in an irregular channel with varying mass diffusion, International journal of heat and mass transfer 55 (11-12) (2012) 3076–3089.

[21] D. Dey, B. Chutia, Dusty nanofluid flow with bioconvection past a vertical stretching surface, Journal of King Saud University-Engineering Sciences 34 (6) (2022) 375–380.

[22] S. U. Rehman, N. Fatima, B. Ali, M. Imran, L. Ali, N. A. Shah, J. D. Chung, The casson dusty nanofluid: Significance of darcy–forchheimer law, magnetic field, and non-fourier heat flux model subject to stretch surface, Mathematics 10 (16) (2022) 2877.

[23] A. Hussain, M. Arshad, A. Rehman, A. Hassan, S. Elagan, H. Ahmad, A. Ishan, Three-dimensional water-based magneto-hydrodynamic rotating nanofluid flow over a linear extending sheet and heat transport analysis: A numerical approach, Energies 14 (16) (2021) 5133.

[24] N. S. Khan, Q. Shah, A. Bhaumik, P. Kumam, P. Thounthong, I. Amiri, Entropy generation in bioconvection nanofluid flow between two stretchable rotating disks, Scientific reports 10 (1) (2020) 4448.

[25] R. Nazar, N. Amin, I. Pop, Unsteady boundary layer flow due to a stretching surface in a rotating fluid, Mechanics Research Communications 31 (1) (2004) 121–128.

[26] A. Ali Zafar, M. Bilal Riaz, M. Imran Asjad, Unsteady rotational flow of fractional maxwell fluid in a cylinder subject to shear stress on the boundary, Punjab University Journal of Mathematics 50 (2).

[27] M. Hussain, H. Sharif, M. A. Khadimallah, H. Ayed, E. M. Banoqitah, H. Loukil, I. Ali, S. Mahmoud, A. Tounsi, Use of rotating disk for darcy-forchheimer flow of nanofluid; similarity transformation through porous media, COMPUTERS AND CONCRETE 30 (1) (2022) 1–8.

[28] X. Liu, Y. Sun, Y. Morisada, H. Fujii, Dynamics of rotational flow in friction stir welding of aluminium alloys, Journal of Materials Processing Technology 252 (2018) 643–651.

[29] A. Hussain, M. H. Alshbool, A. Abdussattar, A. Rehman, H. Ahmad, T. A. Nofal, M. R. Khan, A computational model for hybrid nanofluid flow on a rotating surface in the existence of convective condition, Case Studies in Thermal Engineering 26 (2021) 101089.

[30] M. Ramzan, S. Riasat, J. D. Chung, Y.-M. Chu, M. Sheikholeslami, S. Kadry, F. Howari, Upshot of heterogeneous catalysis in a nanofluid flow over a rotating disk with slip effects and entropy optimization analysis, Scientific Reports 11 (1) (2021) 120.

[31] H. Alotaibi, K. Rafique, Numerical analysis of micro-rotation effect on nanofluid flow for vertical riga plate, Crystals 11 (11) (2021) 1315.

[32] B. Ali, A. K. Hussein, S. Hussain, R. A. Naqvi, et al., Transient rotating nanofluid flow over a riga plate with gyrotactic micro-organisms, binary chemical reaction and non-fourier heat flux, Chinese Journal of Physics 73 (2021) 732–745.

[33] S. E. Ahmed, A. A. Arafa, S. A. Hussein, Z. A. Raizah, Novel treatments for the bioconvective radiative ellis nanofluids wedge flow with viscous dissipation and an activation energy, Case Studies in Thermal Engineering 40 (2022) 102510.

[34] M. Jalil, S. Asghar, S. Yasmeen, An exact solution of mhd boundary layer flow of dusty fluid over a stretching surface, Mathematical Problems in Engineering 2017 (2017) 1–5.

[35] A. U. Awan, N. A. Ahammad, S. Majeed, F. Gamaoun, B. Ali, Significance of hybrid nanoparticles, lorentz and coriolis forces on the dynamics of water based flow, International Communications in Heat and Mass Transfer 135 (2022) 106084.

[36] R. Manghat, S. Siddabasappa, Mhd boundary layer flow and heat transfer of rotating dusty nanofluid over a stretching surface, Kyungpook Mathematical Journal 60 (4) (2020) 853–867.

[37] B. Souayeh, Simultaneous features of cc heat flux on dusty ternary nanofluid (graphene+ tungsten oxide+ zirconium oxide) through a magnetic field with slippery condition, Mathematics 11 (3) (2023) 554.

[38] Y. Wei, S. U. Rehman, N. Fatima, B. Ali, L. Ali, J. D. Chung, N. A. Shah, Significance of dust particles, nanoparticles radius, coriolis and lorentz forces: The case of maxwell dusty fluid, Nanomaterials 12 (9) (2022) 1512.

[39] S. U. Rehman, A. Mariam, A. Ullah, M. I. Asjad, M. Y. Bajuri, B. A. Pansera, A. Ahmadian, Numerical computation of buoyancy and radiation effects on mhd micropolar nanofluid flow over a stretching/shrinking sheet with heat source, Case Studies in Thermal Engineering 25 (2021) 100867.

[40] Q. Lou, B. Ali, S. U. Rehman, D. Habib, S. Abdal, N. A. Shah, J. D. Chung, Micropolar dusty fluid: Coriolis force effects on dynamics of mhd rotating fluid when lorentz force is significant, Mathematics 10 (15) (2022) 2630.
