# Supplementary figures and images for: Numerical investigation of dusty tri-hybrid Ellis rotating nanofluid flow and thermal transportation over a stretchable Riga plate
Source: Sci Rep. 2023 Aug 31;13:14272. doi: 10.1038/s41598-023-41141-1 (PMC10471745; doi:10.1038/s41598-023-41141-1)

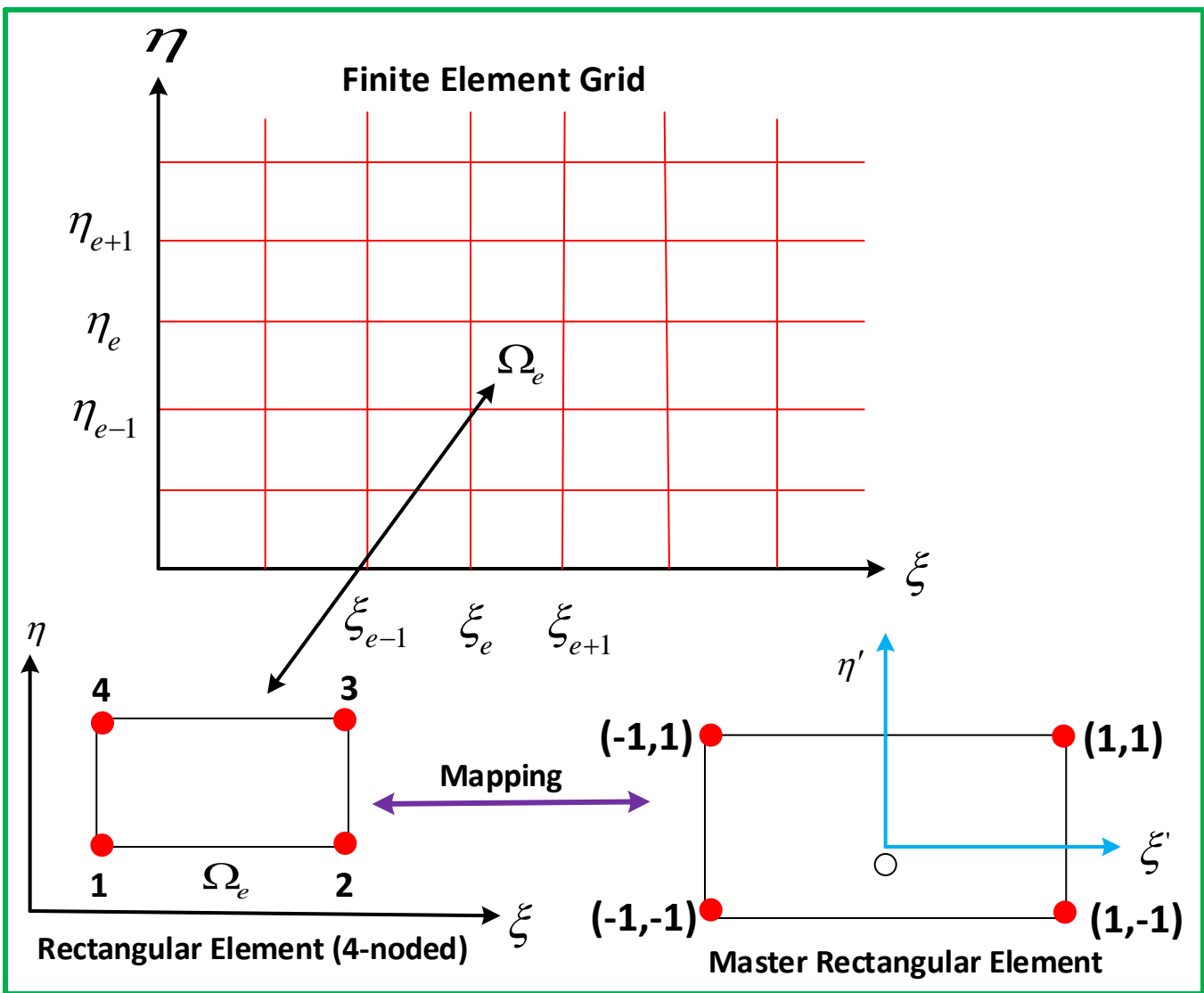

Supplement: Supplementary file 1 — Supplementary Information. [file 41598_2023_41141_MOESM1_ESM.zip › P22- dusty Ellis tri/2D_Grid.pdf]

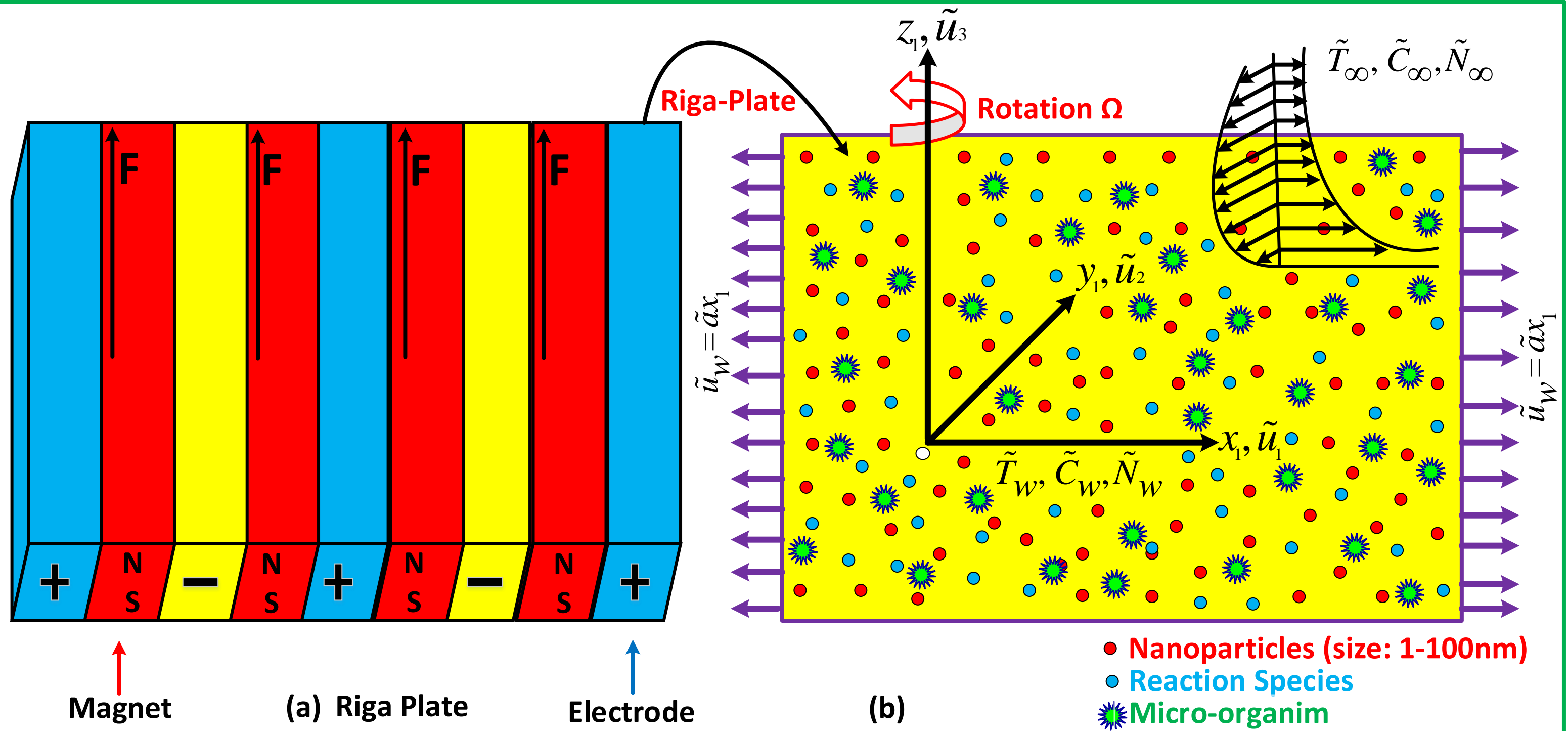

Supplement: Supplementary file 1 — Supplementary Information. [file 41598_2023_41141_MOESM1_ESM.zip › P22- dusty Ellis tri/3D_Model_Riga.pdf]

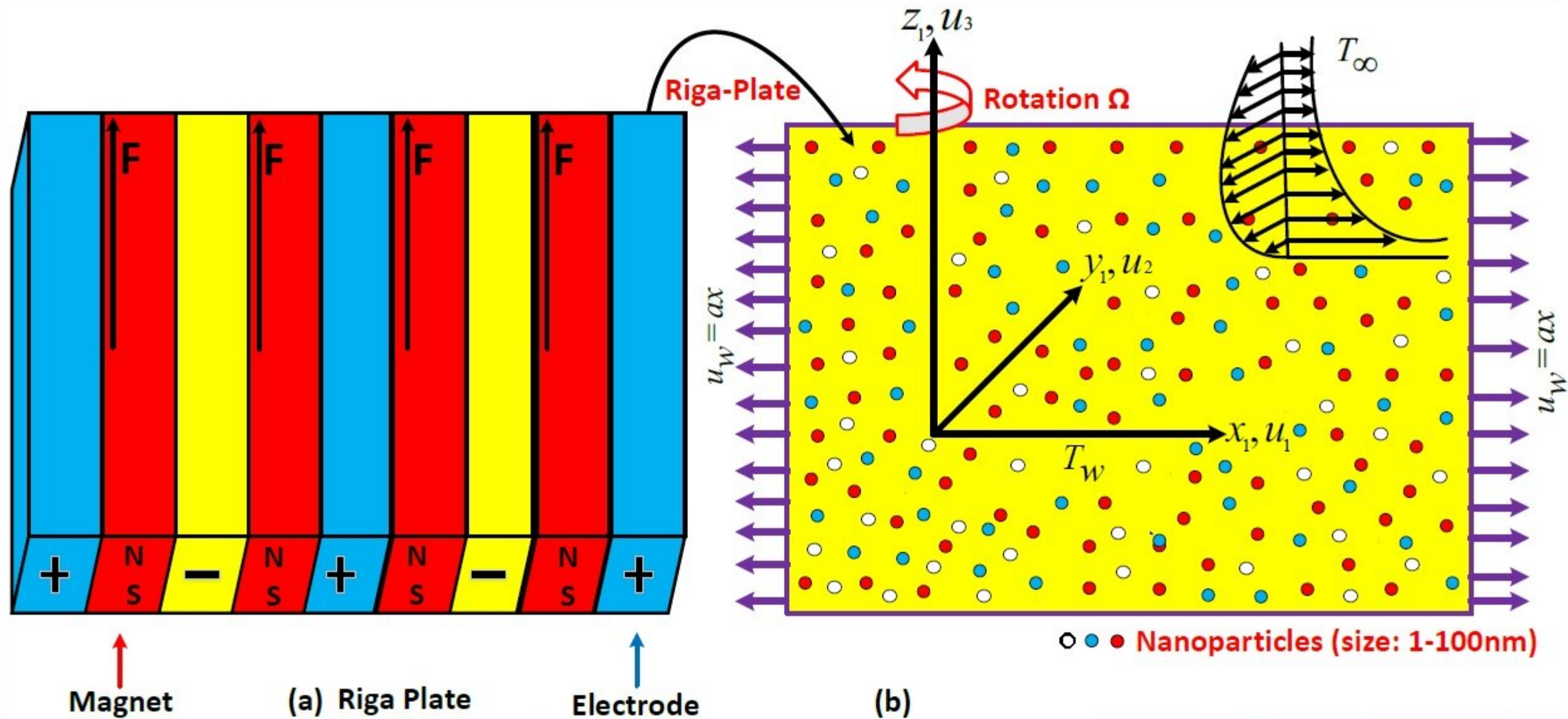

Supplement: Supplementary file 1 — Supplementary Information. [file 41598_2023_41141_MOESM1_ESM.zip › P22- dusty Ellis tri/3driga.pdf]

(a)

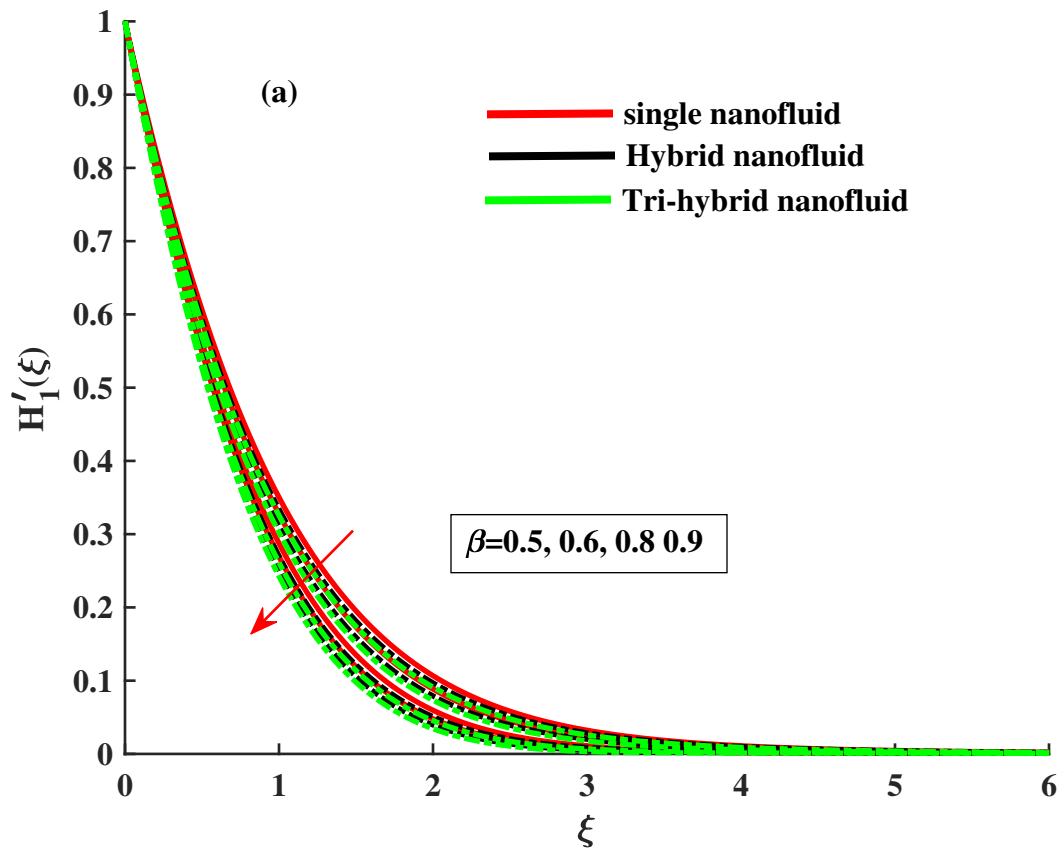

Supplement: Supplementary file 1 — Supplementary Information. [file 41598_2023_41141_MOESM1_ESM.zip › P22- dusty Ellis tri/betaf-eps-converted-to.pdf]

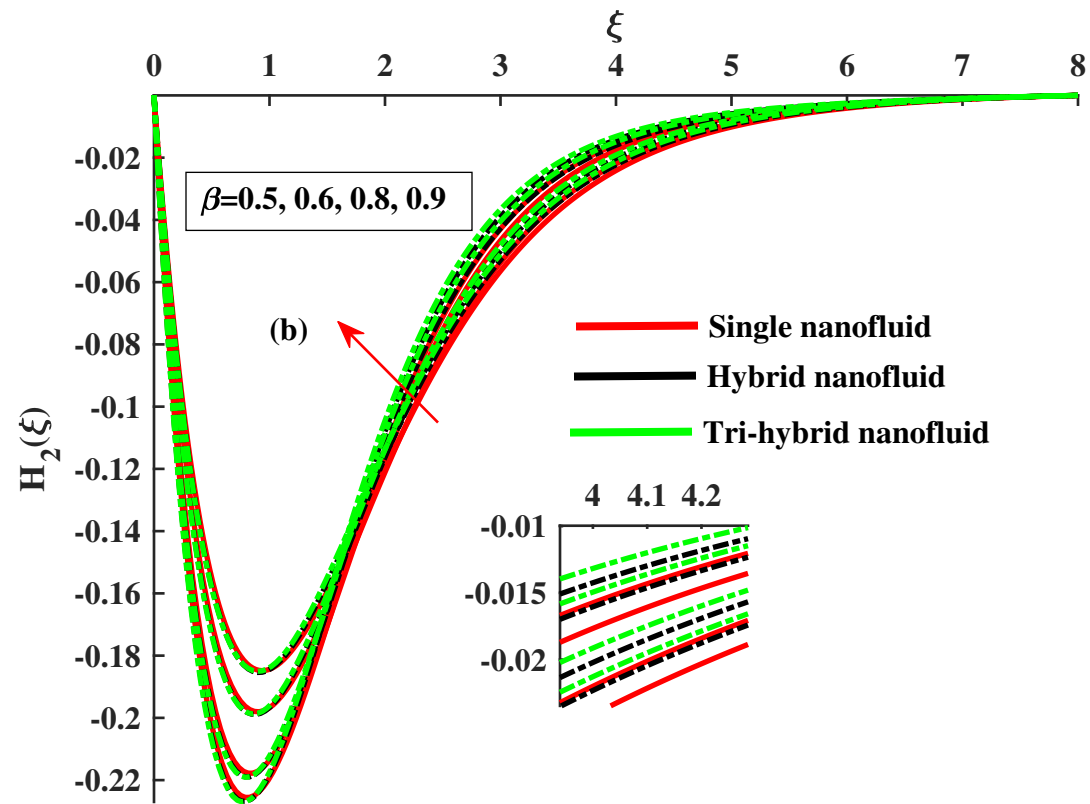

Supplement: Supplementary file 1 — Supplementary Information. [file 41598_2023_41141_MOESM1_ESM.zip › P22- dusty Ellis tri/betag-eps-converted-to.pdf]

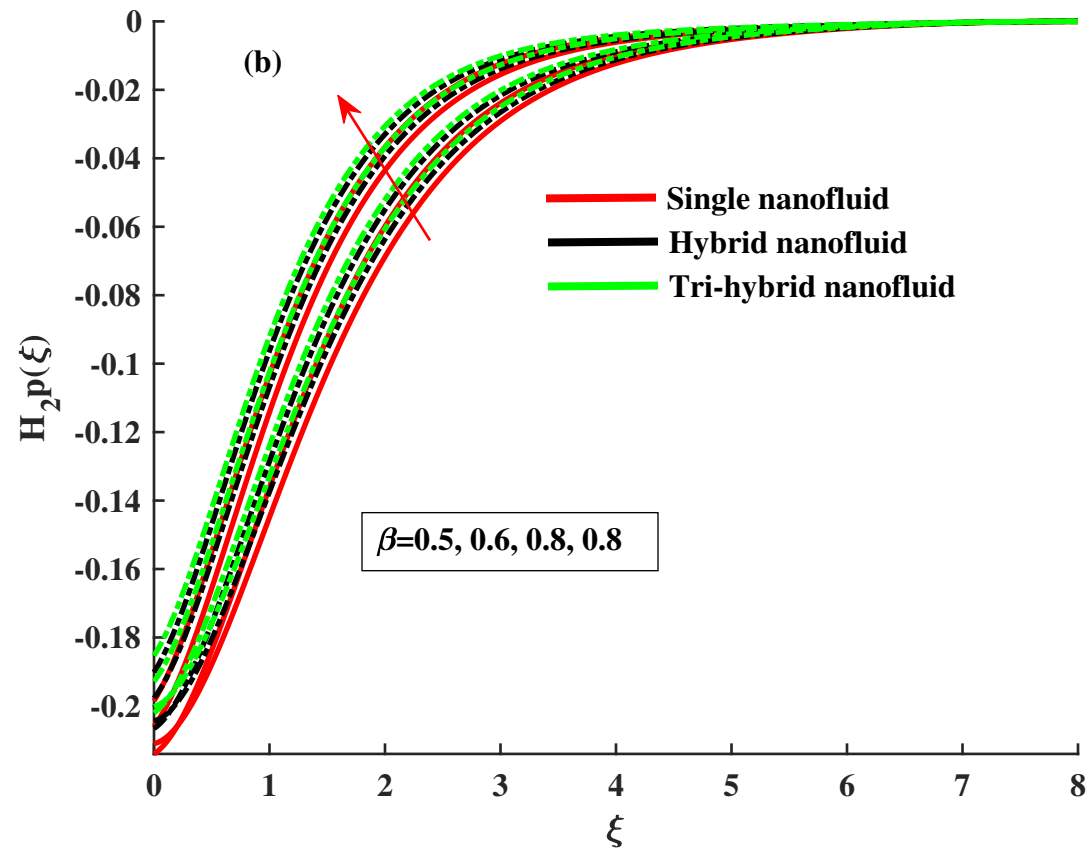

Supplement: Supplementary file 1 — Supplementary Information. [file 41598_2023_41141_MOESM1_ESM.zip › P22- dusty Ellis tri/betaH2P-eps-converted-to.pdf]

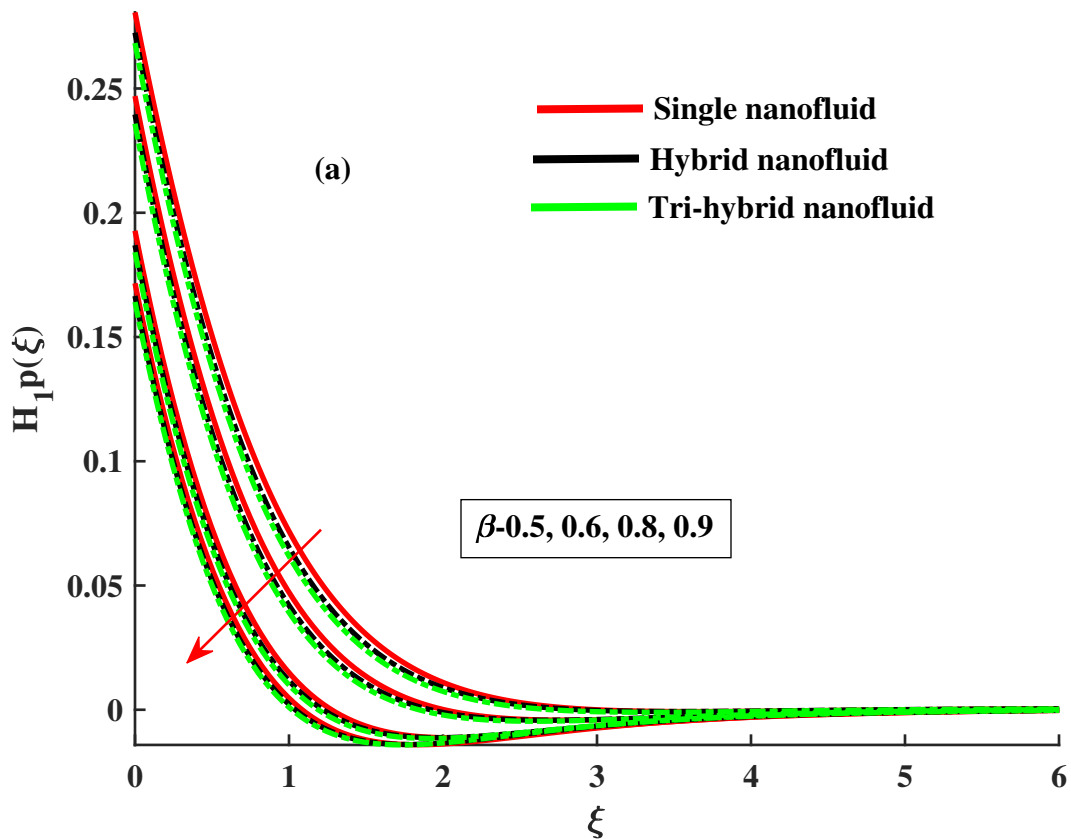

Supplement: Supplementary file 1 — Supplementary Information. [file 41598_2023_41141_MOESM1_ESM.zip › P22- dusty Ellis tri/betaHp-eps-converted-to.pdf]

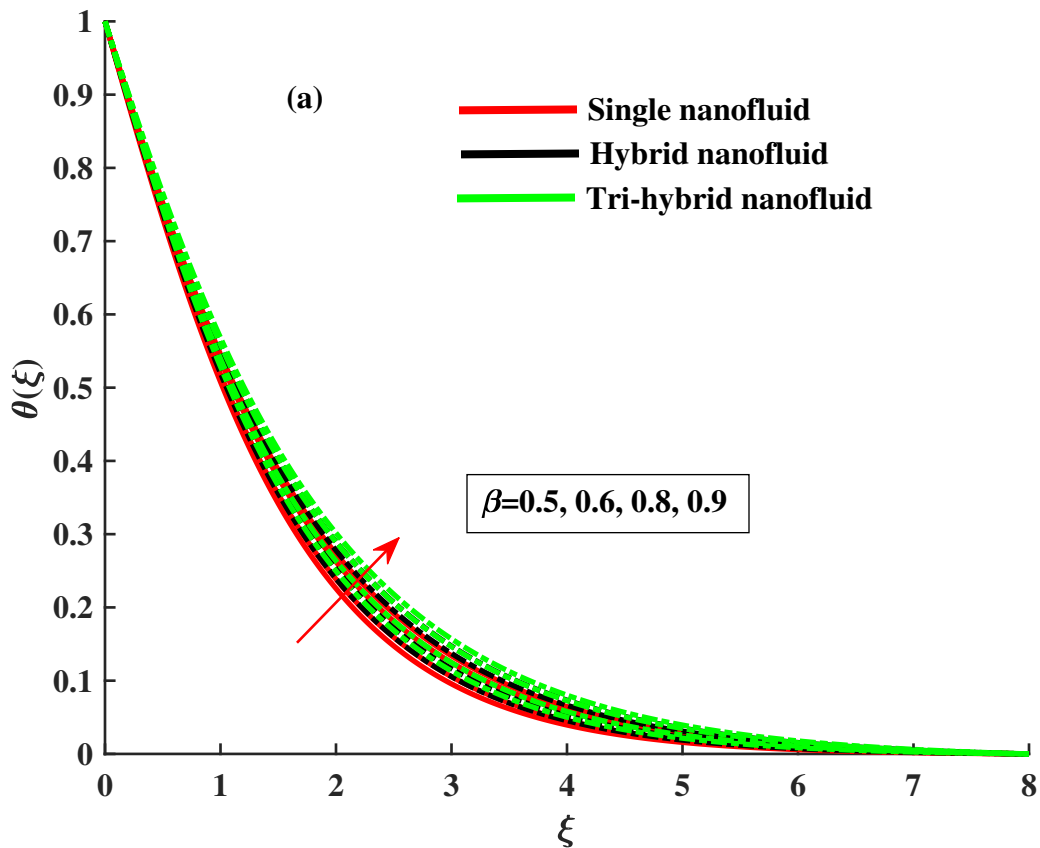

Supplement: Supplementary file 1 — Supplementary Information. [file 41598_2023_41141_MOESM1_ESM.zip › P22- dusty Ellis tri/betathta-eps-converted-to.pdf]

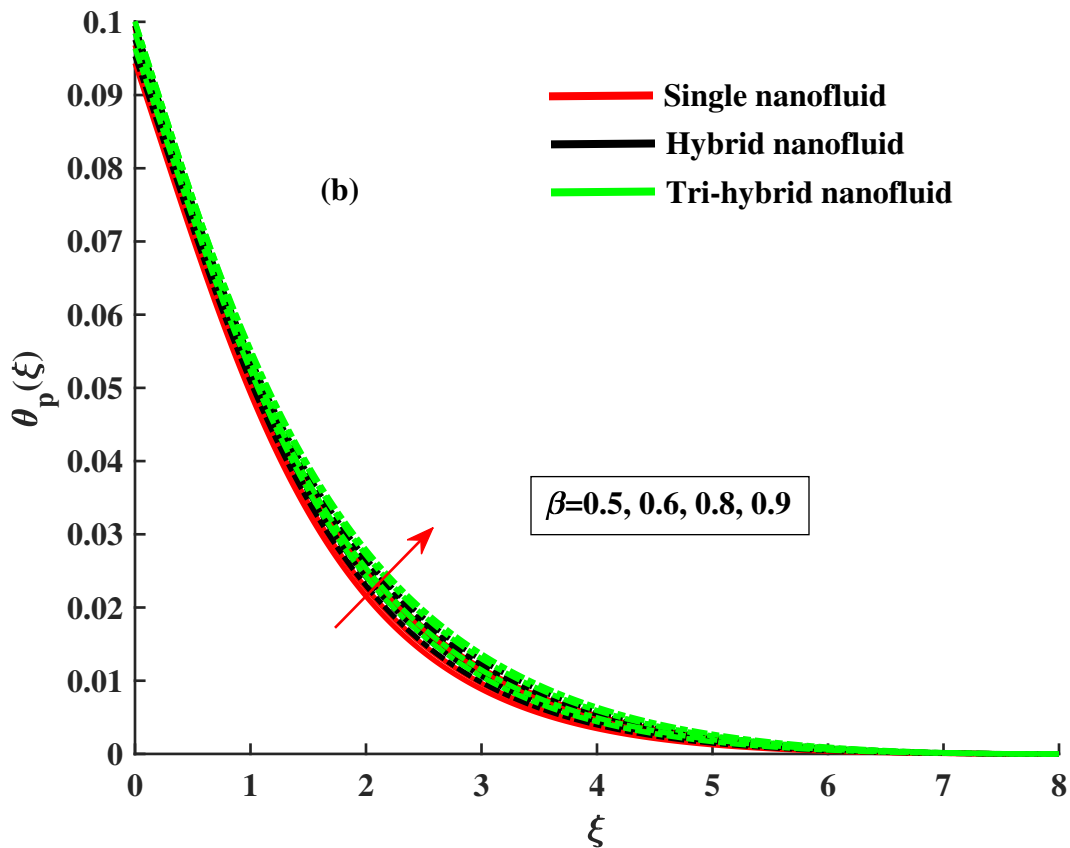

Supplement: Supplementary file 1 — Supplementary Information. [file 41598_2023_41141_MOESM1_ESM.zip › P22- dusty Ellis tri/betathtap-eps-converted-to.pdf]

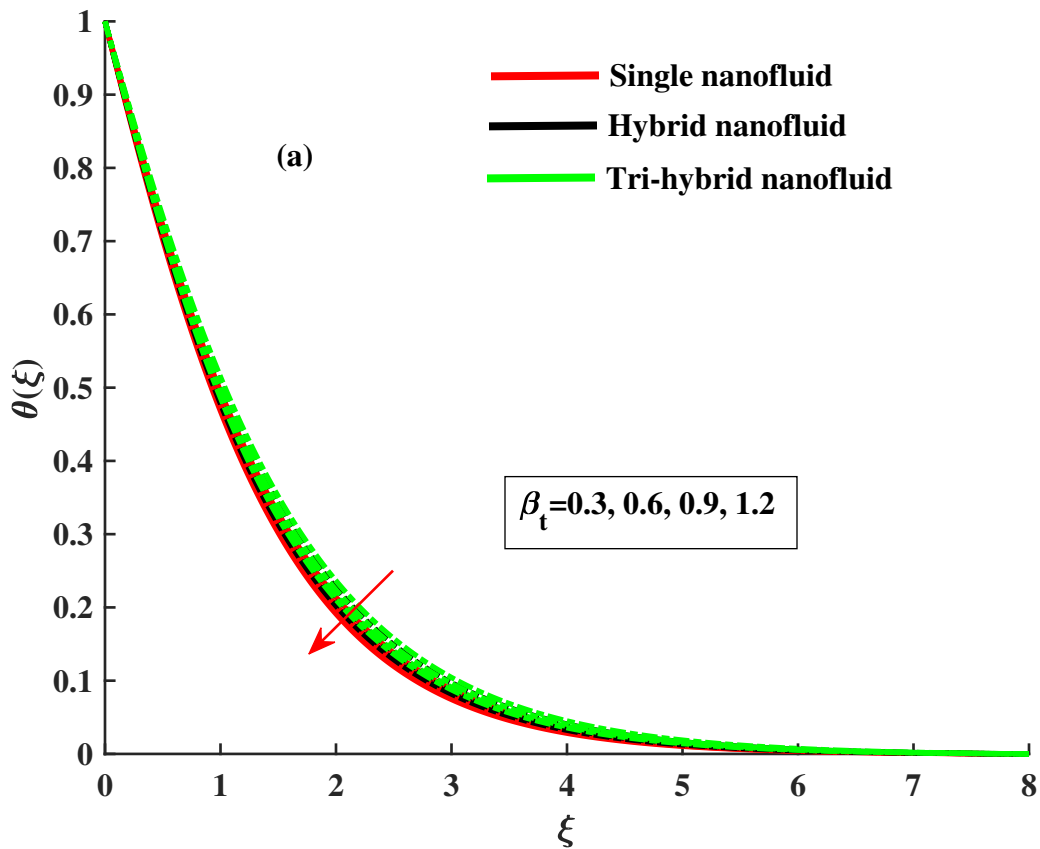

Supplement: Supplementary file 1 — Supplementary Information. [file 41598_2023_41141_MOESM1_ESM.zip › P22- dusty Ellis tri/betatthta-eps-converted-to.pdf]

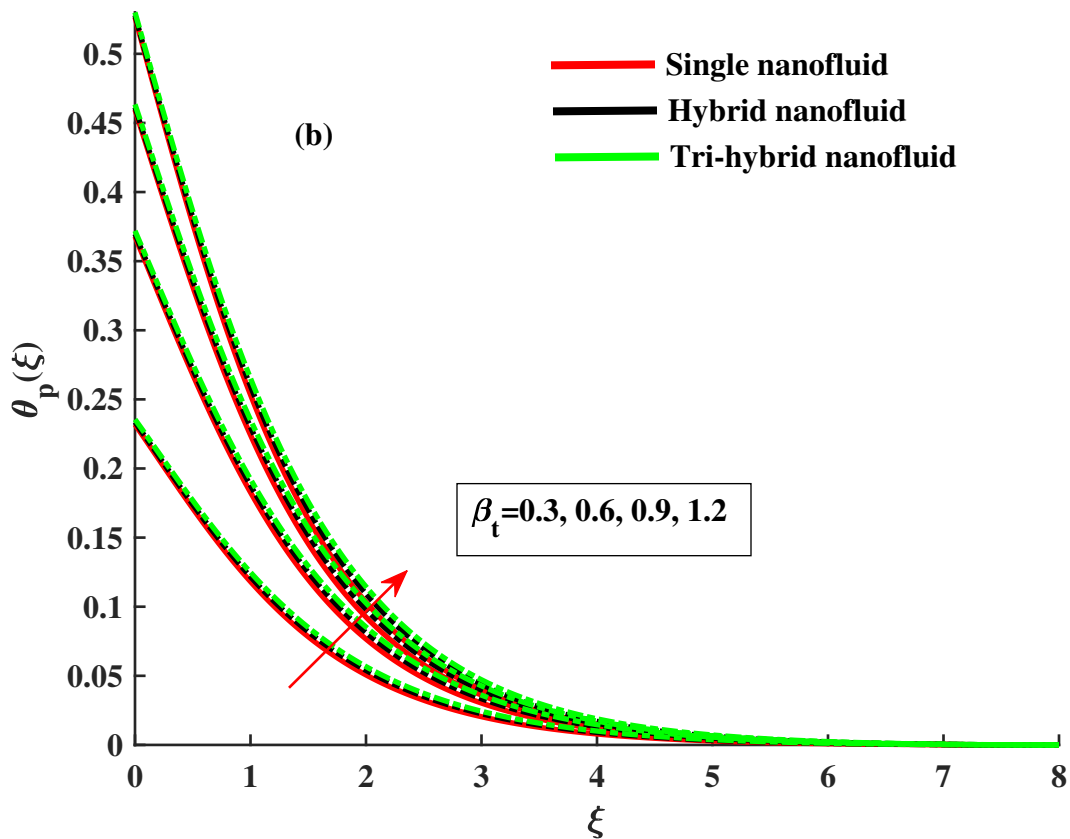

Supplement: Supplementary file 1 — Supplementary Information. [file 41598_2023_41141_MOESM1_ESM.zip › P22- dusty Ellis tri/betatthtap-eps-converted-to.pdf]

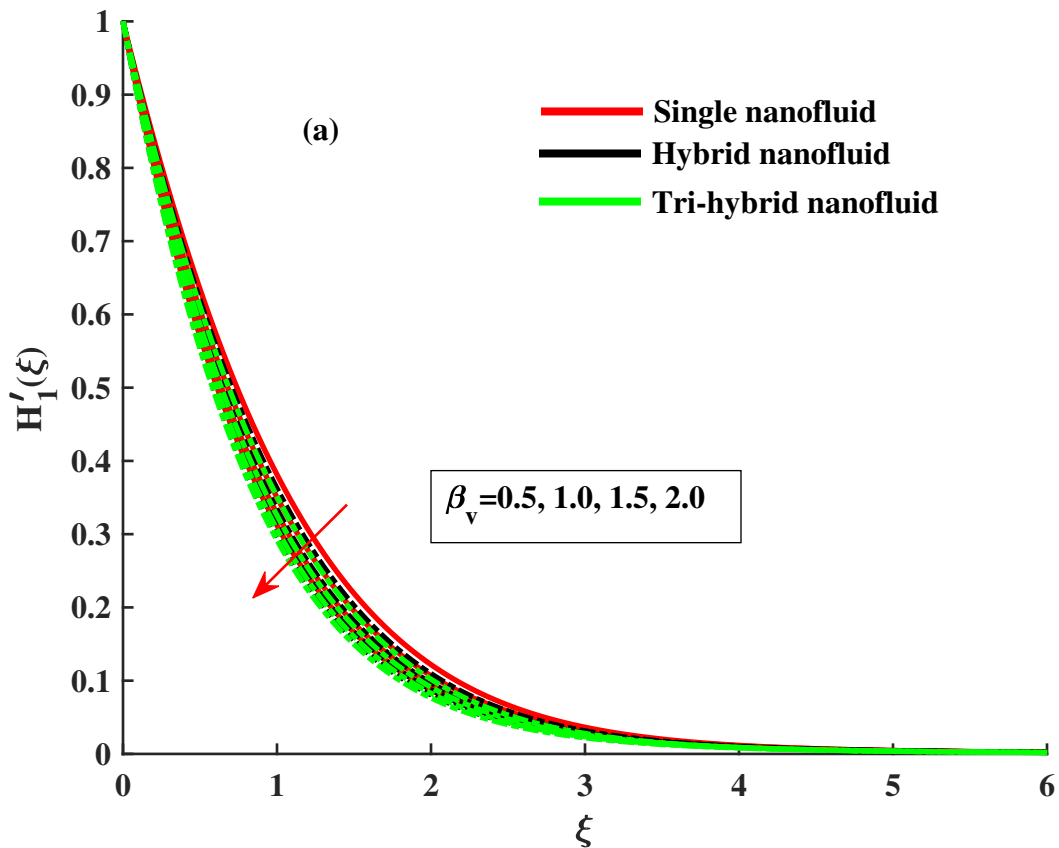

Supplement: Supplementary file 1 — Supplementary Information. [file 41598_2023_41141_MOESM1_ESM.zip › P22- dusty Ellis tri/betavf-eps-converted-to.pdf]

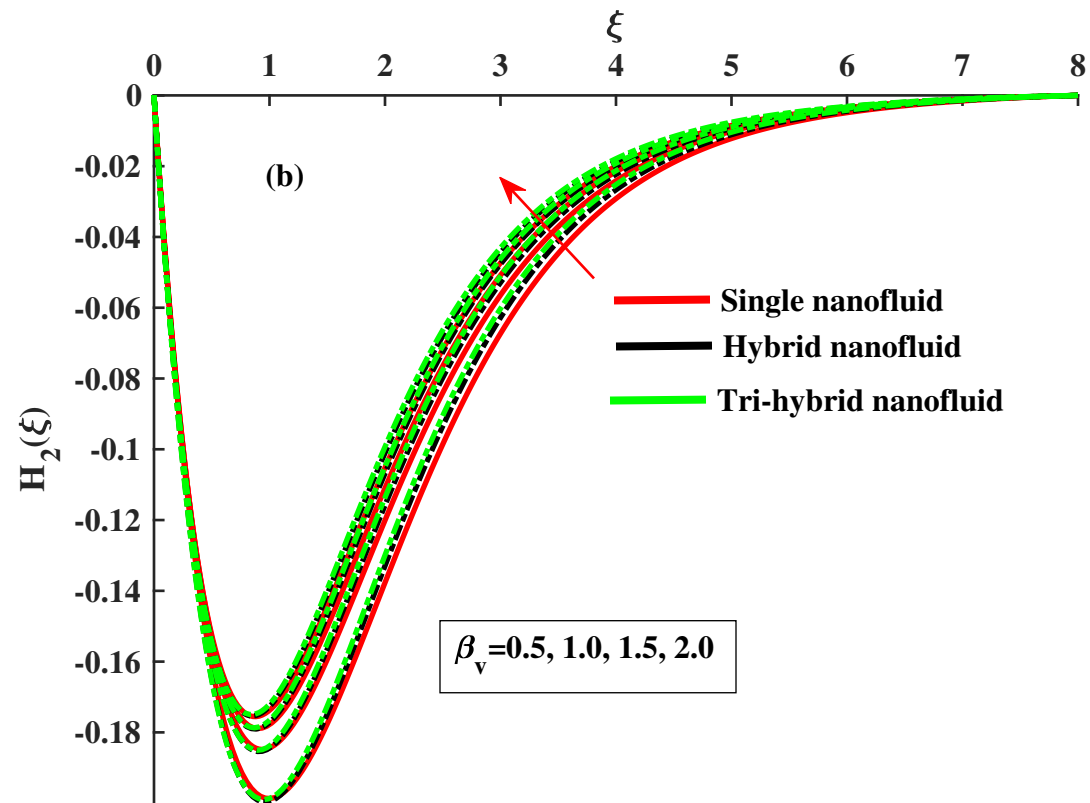

Supplement: Supplementary file 1 — Supplementary Information. [file 41598_2023_41141_MOESM1_ESM.zip › P22- dusty Ellis tri/betavg-eps-converted-to.pdf]

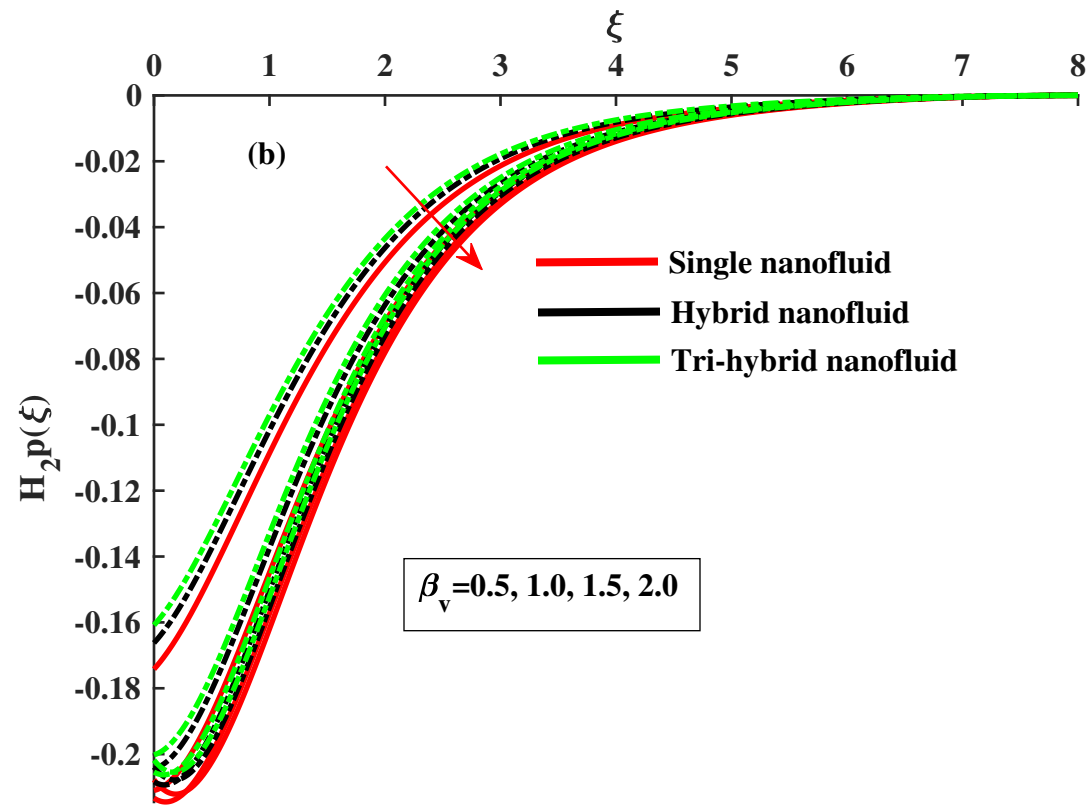

Supplement: Supplementary file 1 — Supplementary Information. [file 41598_2023_41141_MOESM1_ESM.zip › P22- dusty Ellis tri/betavH2p-eps-converted-to.pdf]

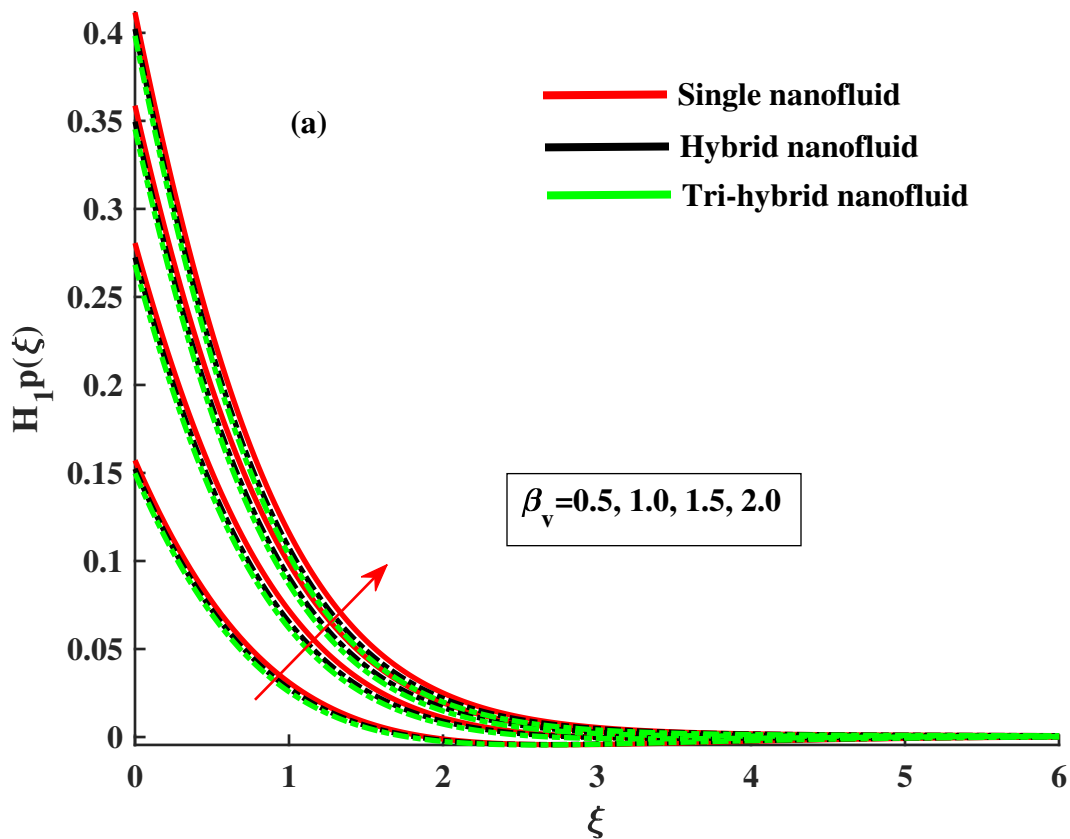

Supplement: Supplementary file 1 — Supplementary Information. [file 41598_2023_41141_MOESM1_ESM.zip › P22- dusty Ellis tri/betavHp-eps-converted-to.pdf]

(a)

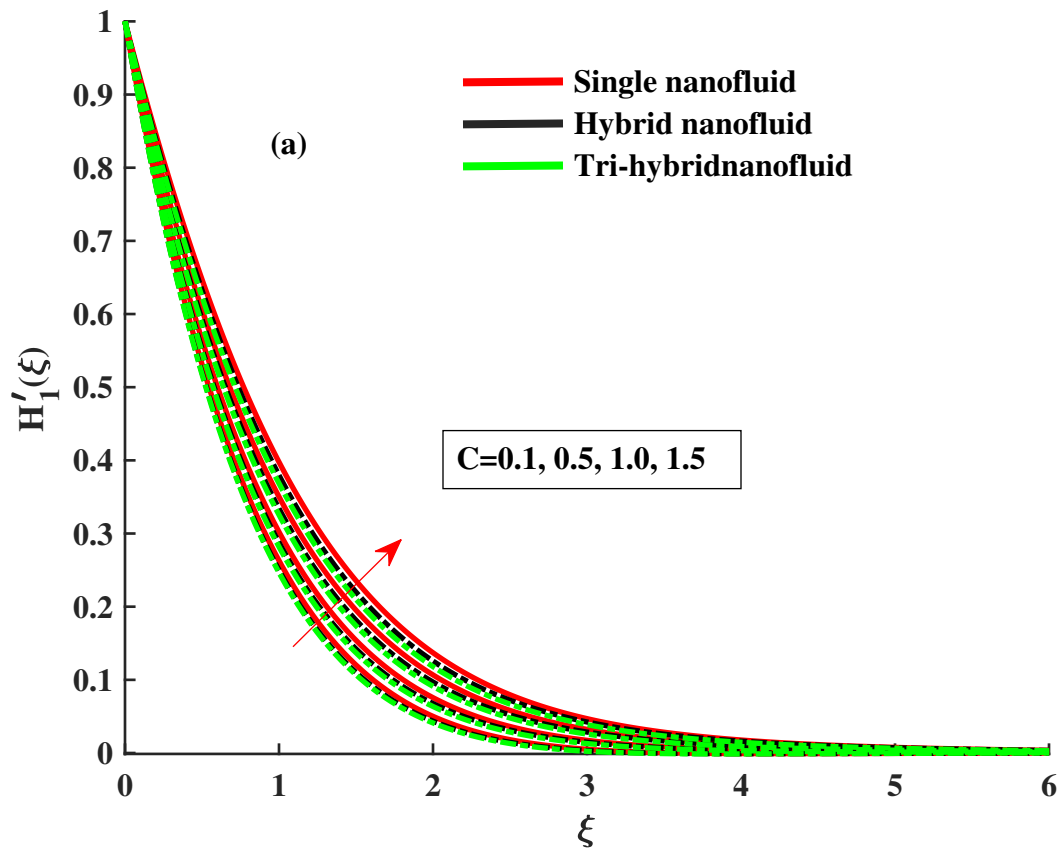

Supplement: Supplementary file 1 — Supplementary Information. [file 41598_2023_41141_MOESM1_ESM.zip › P22- dusty Ellis tri/C1f-eps-converted-to.pdf]

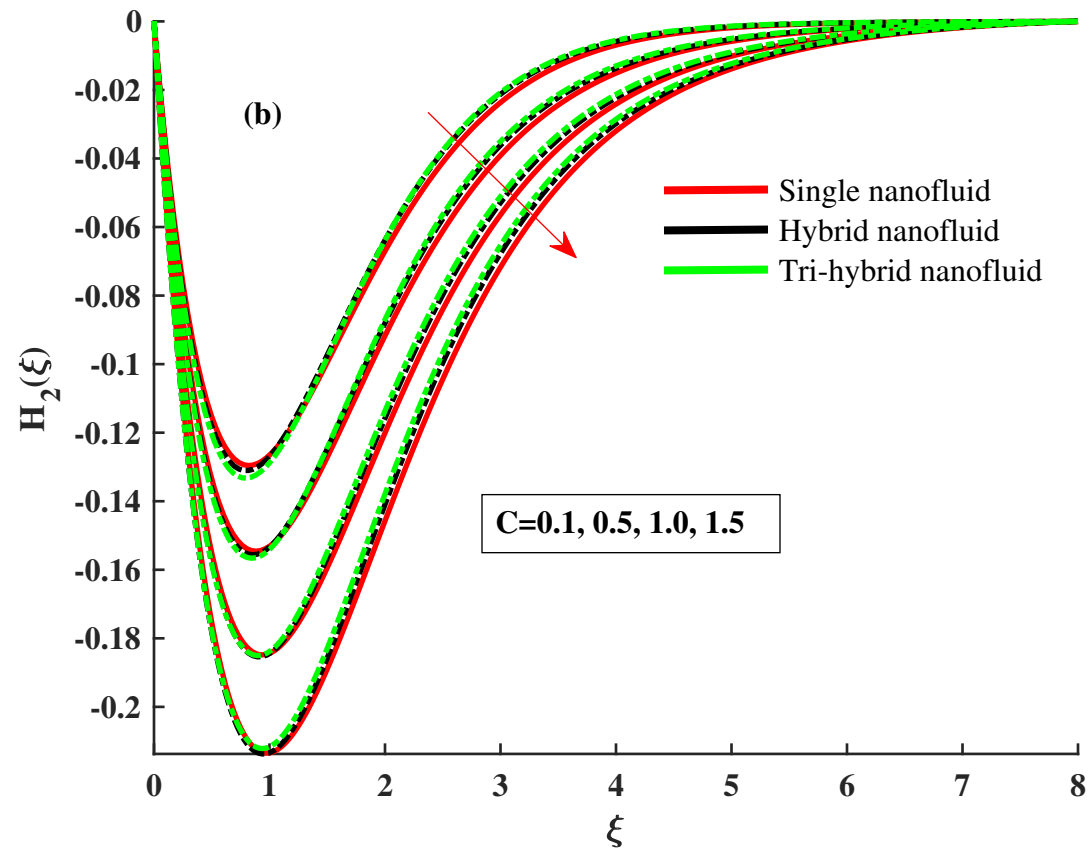

Supplement: Supplementary file 1 — Supplementary Information. [file 41598_2023_41141_MOESM1_ESM.zip › P22- dusty Ellis tri/C1g-eps-converted-to.pdf]

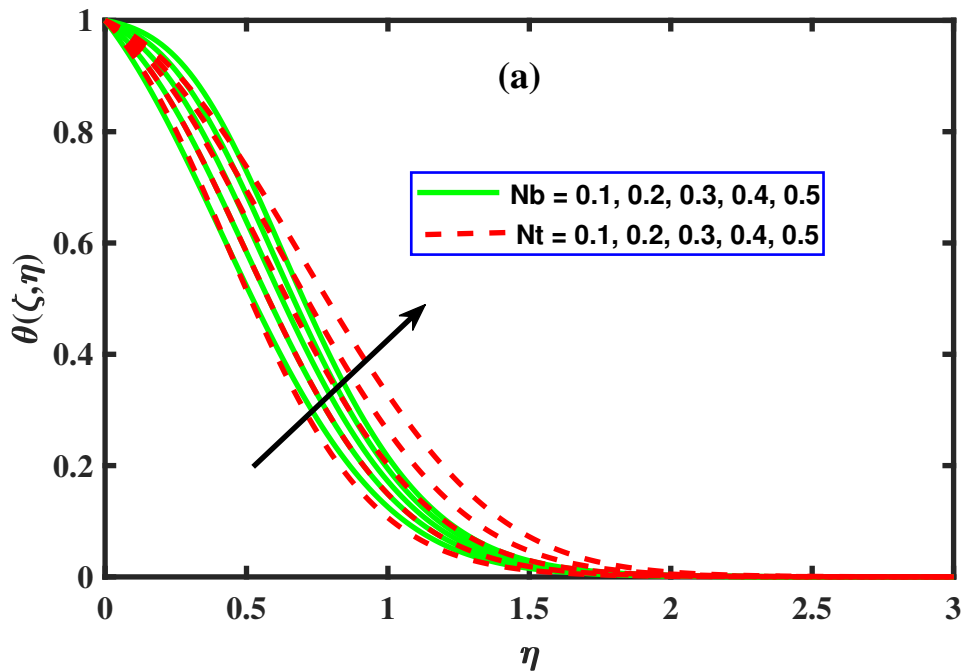

Supplement: Supplementary file 1 — Supplementary Information. [file 41598_2023_41141_MOESM1_ESM.zip › P22- dusty Ellis tri/NbT-eps-converted-to.pdf]

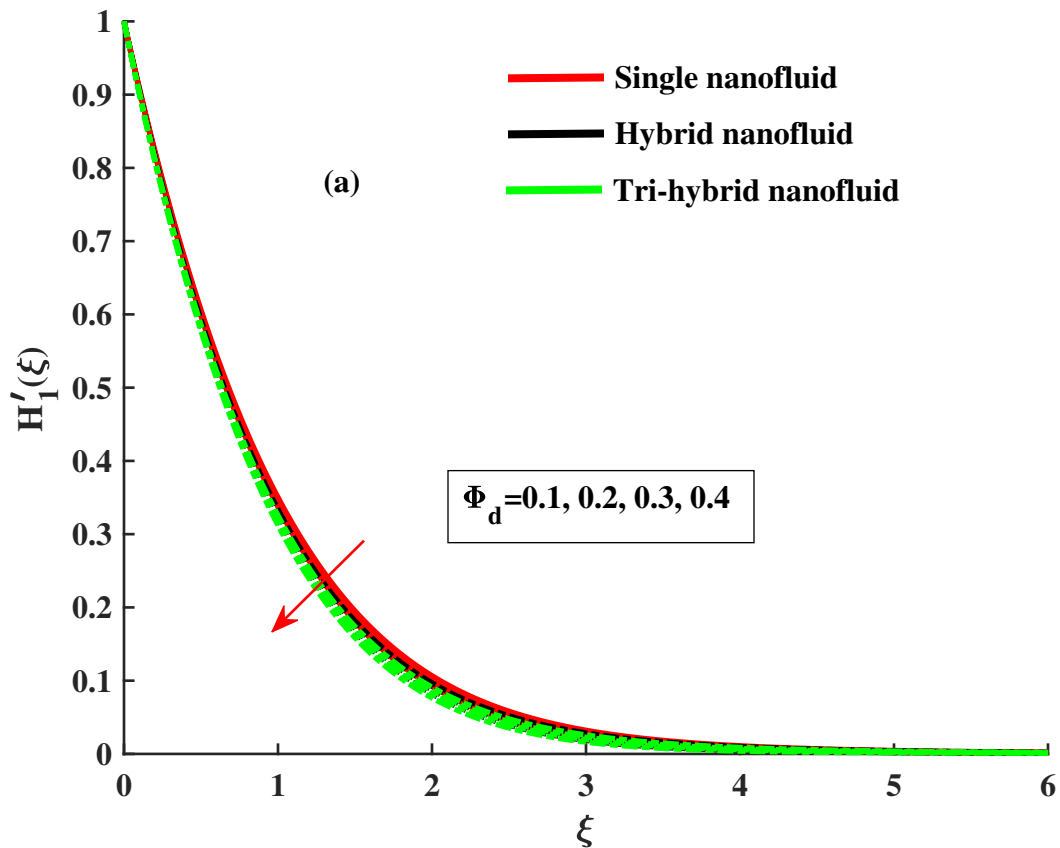

Supplement: Supplementary file 1 — Supplementary Information. [file 41598_2023_41141_MOESM1_ESM.zip › P22- dusty Ellis tri/phidf-eps-converted-to.pdf]

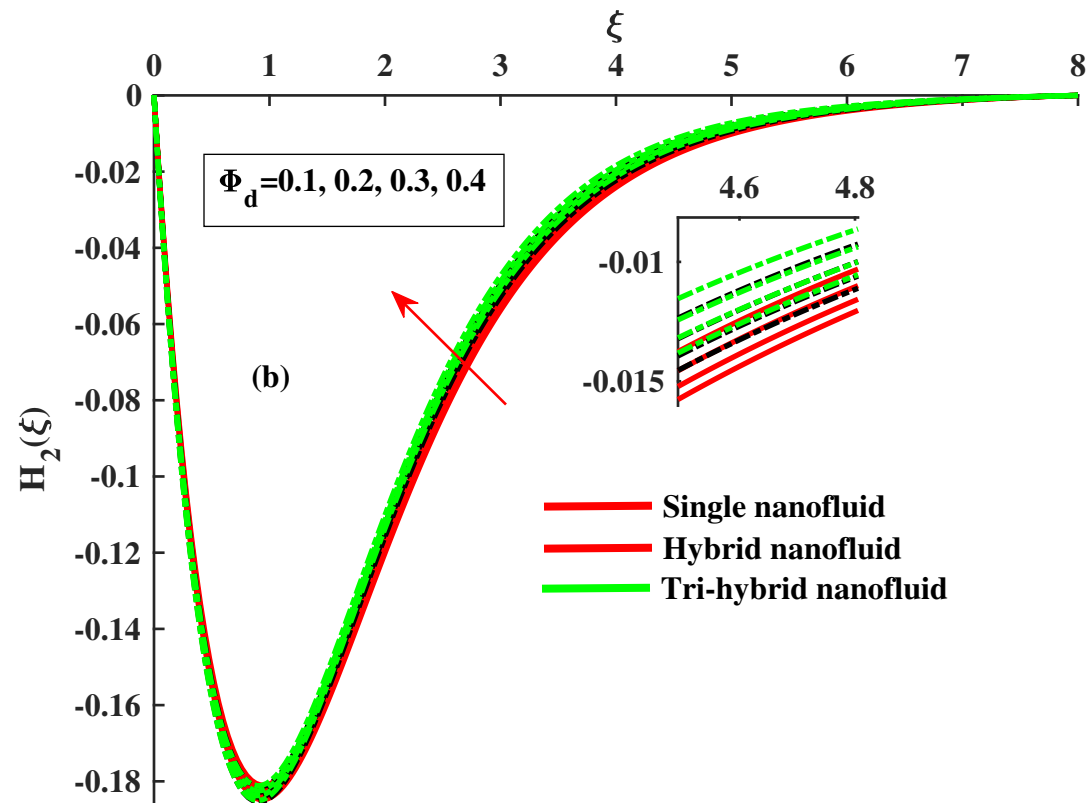

Supplement: Supplementary file 1 — Supplementary Information. [file 41598_2023_41141_MOESM1_ESM.zip › P22- dusty Ellis tri/phidg-eps-converted-to.pdf]

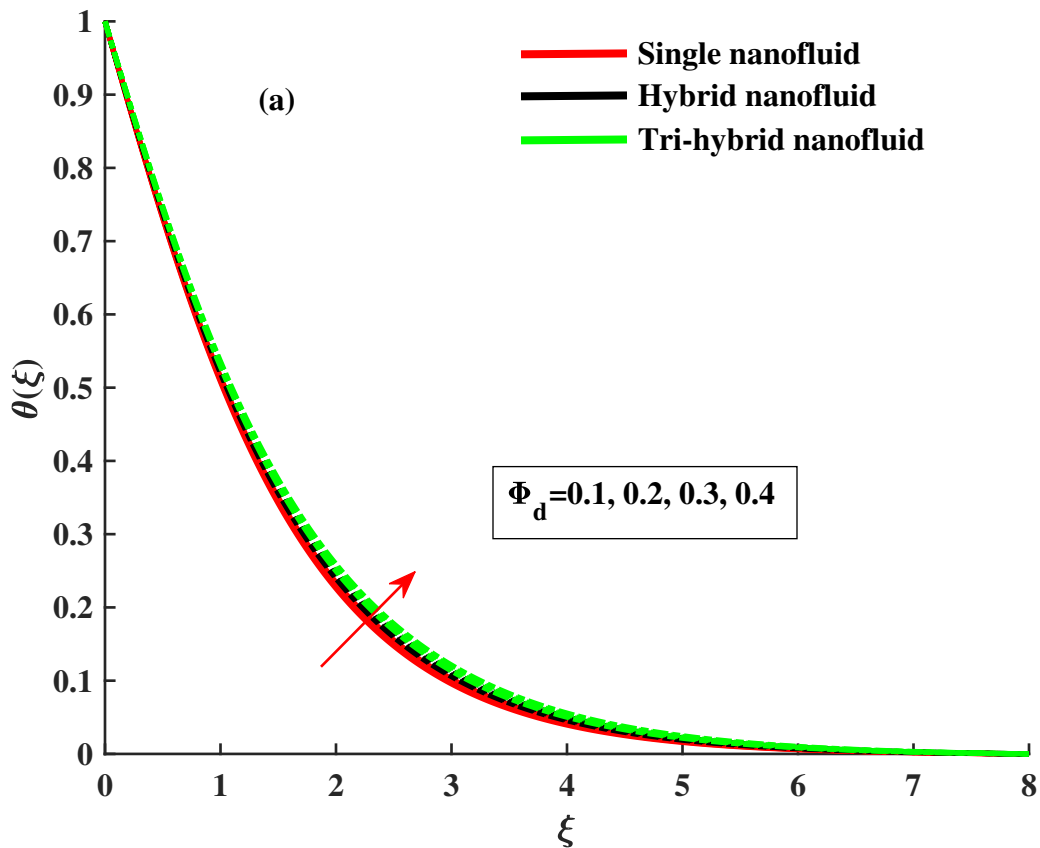

Supplement: Supplementary file 1 — Supplementary Information. [file 41598_2023_41141_MOESM1_ESM.zip › P22- dusty Ellis tri/phidthta-eps-converted-to.pdf]

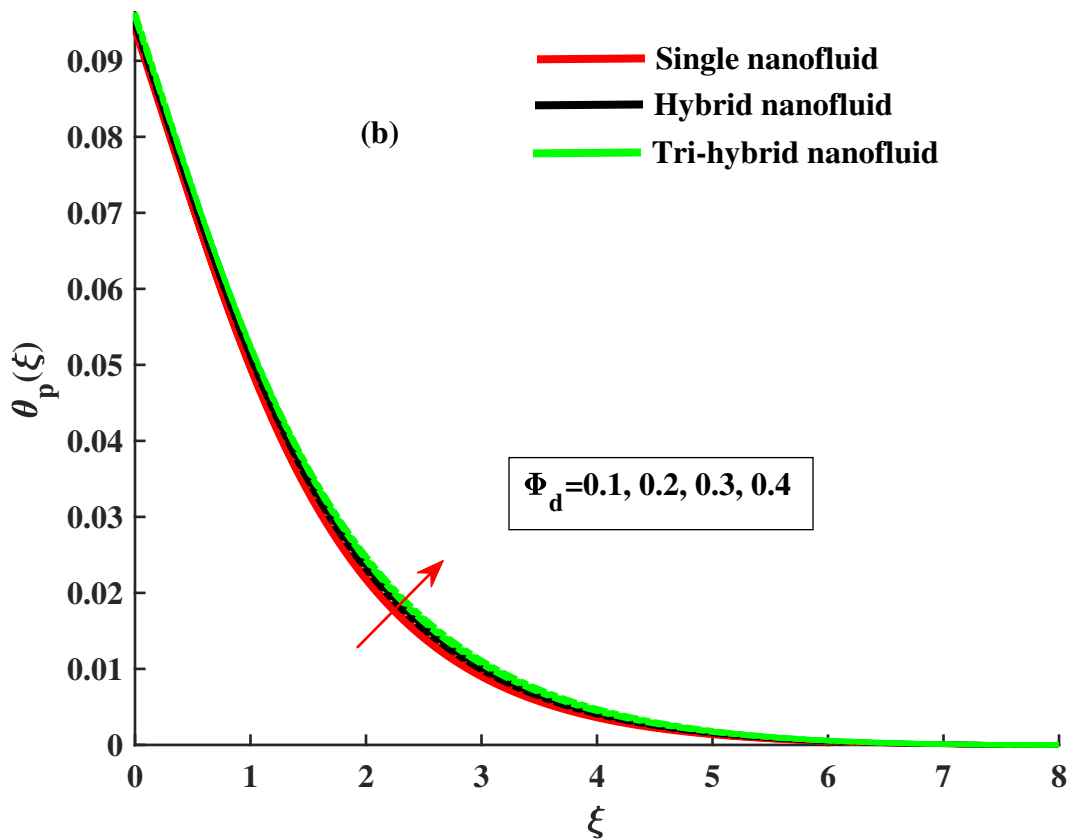

Supplement: Supplementary file 1 — Supplementary Information. [file 41598_2023_41141_MOESM1_ESM.zip › P22- dusty Ellis tri/phidthtap-eps-converted-to.pdf]

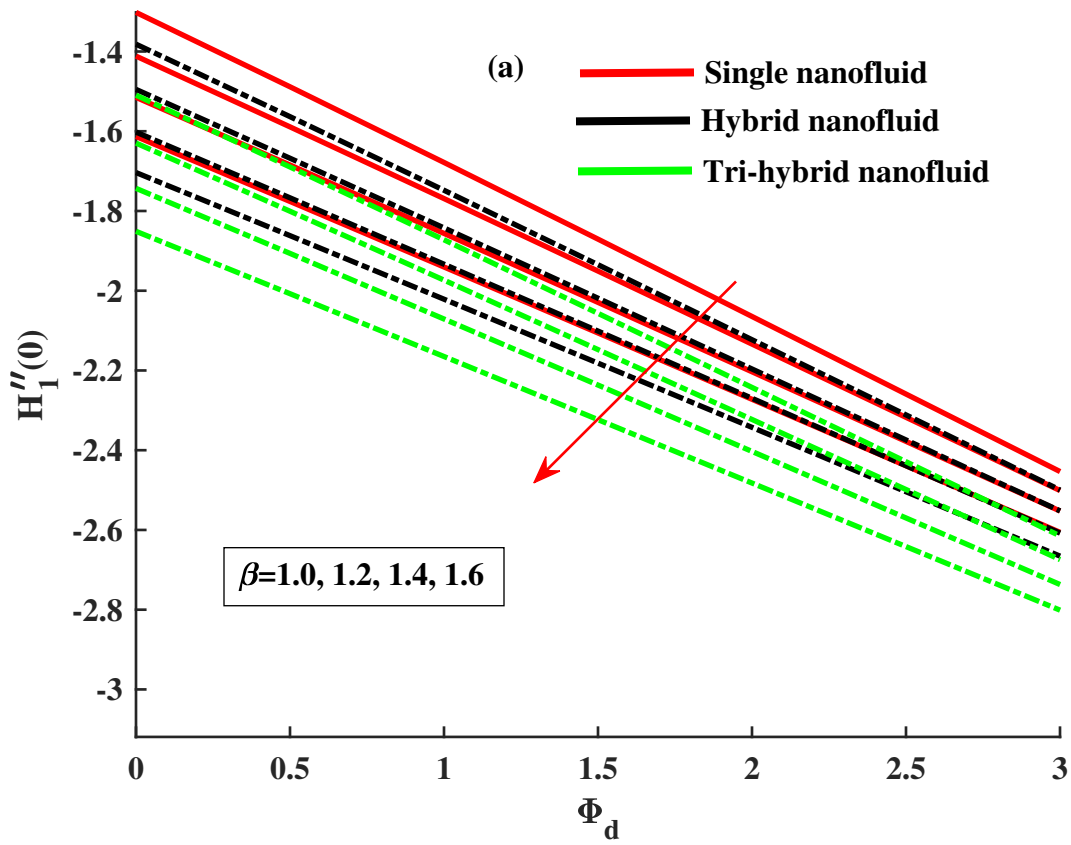

Supplement: Supplementary file 1 — Supplementary Information. [file 41598_2023_41141_MOESM1_ESM.zip › P22- dusty Ellis tri/skinf-eps-converted-to.pdf]

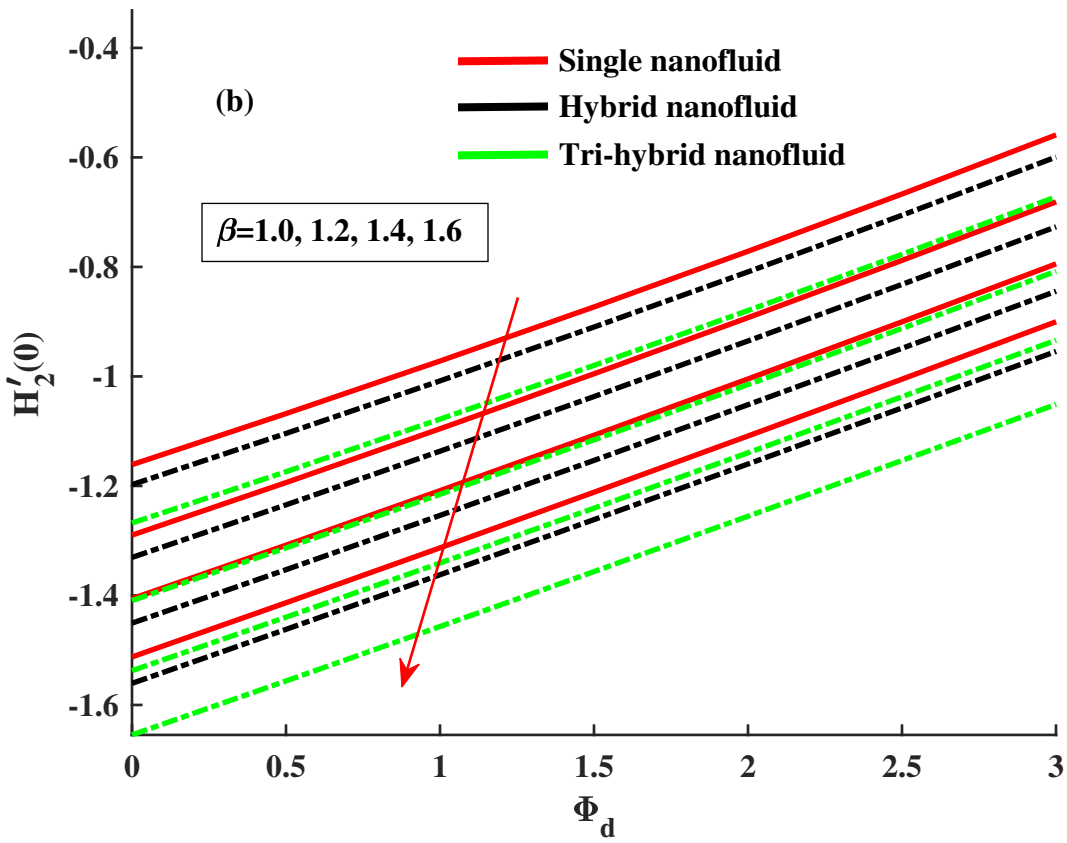

Supplement: Supplementary file 1 — Supplementary Information. [file 41598_2023_41141_MOESM1_ESM.zip › P22- dusty Ellis tri/sking-eps-converted-to.pdf]

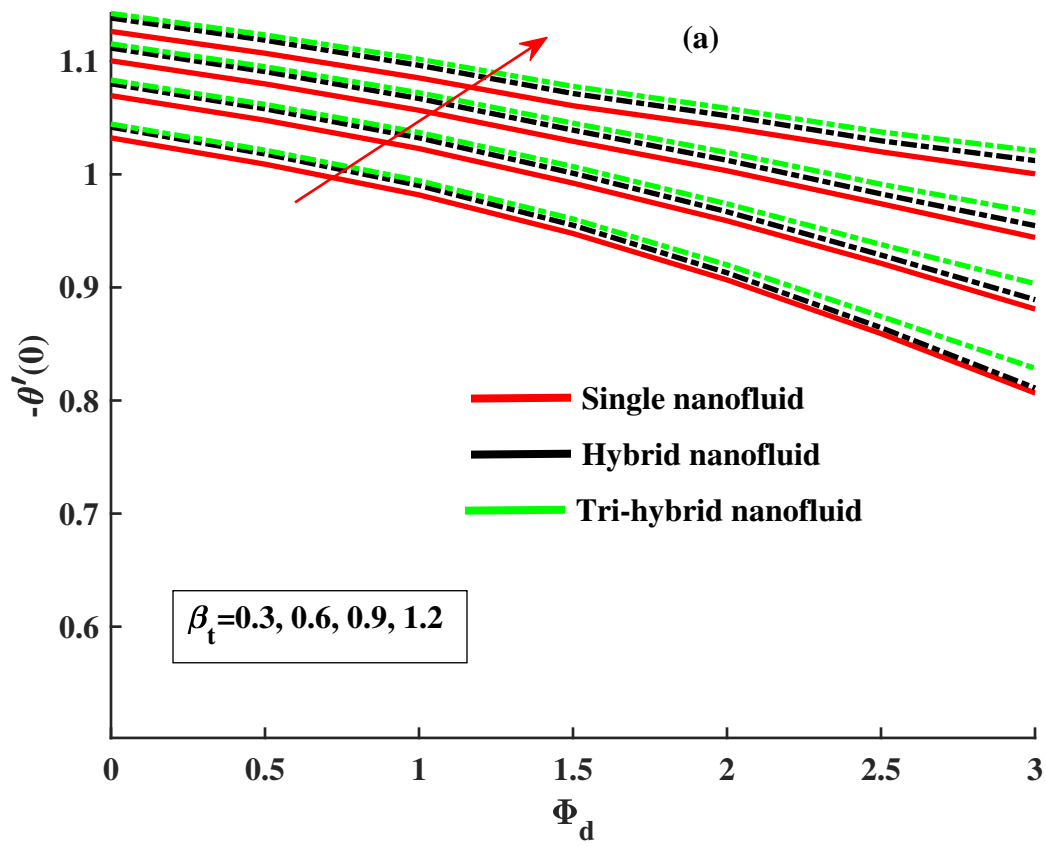

Supplement: Supplementary file 1 — Supplementary Information. [file 41598_2023_41141_MOESM1_ESM.zip › P22- dusty Ellis tri/skinthta1-eps-converted-to.pdf]

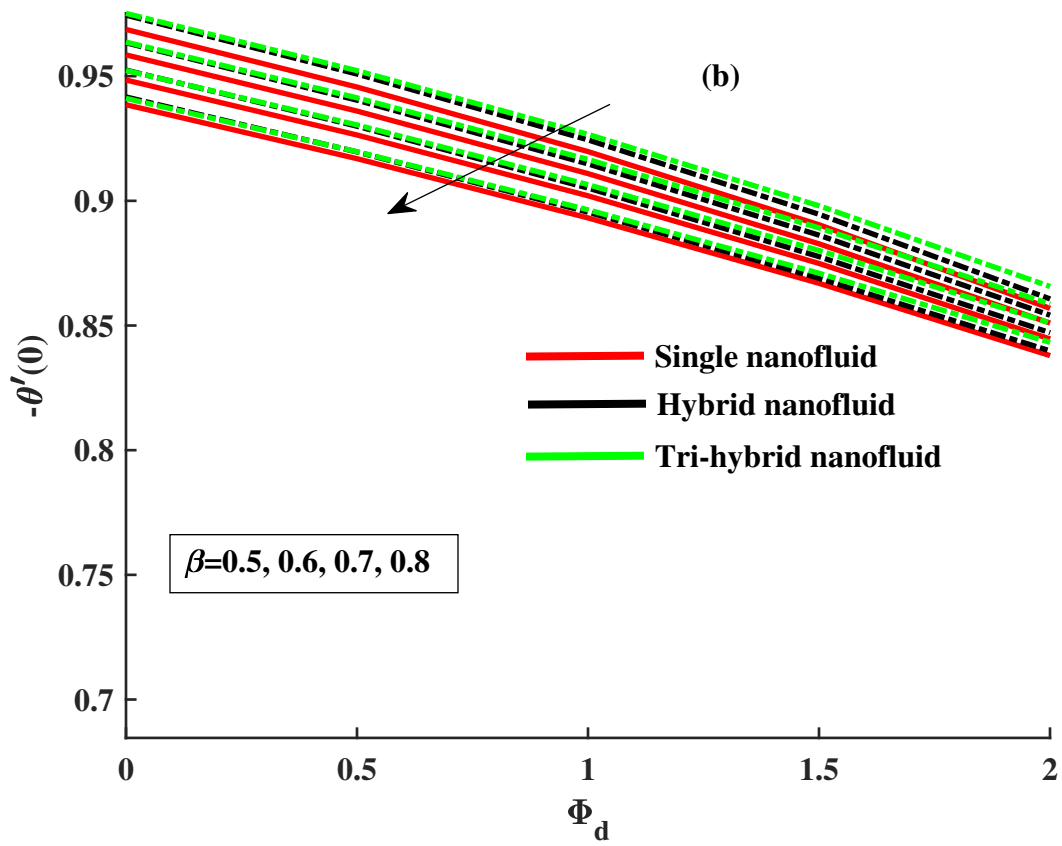

Supplement: Supplementary file 1 — Supplementary Information. [file 41598_2023_41141_MOESM1_ESM.zip › P22- dusty Ellis tri/skinthta2-eps-converted-to.pdf]

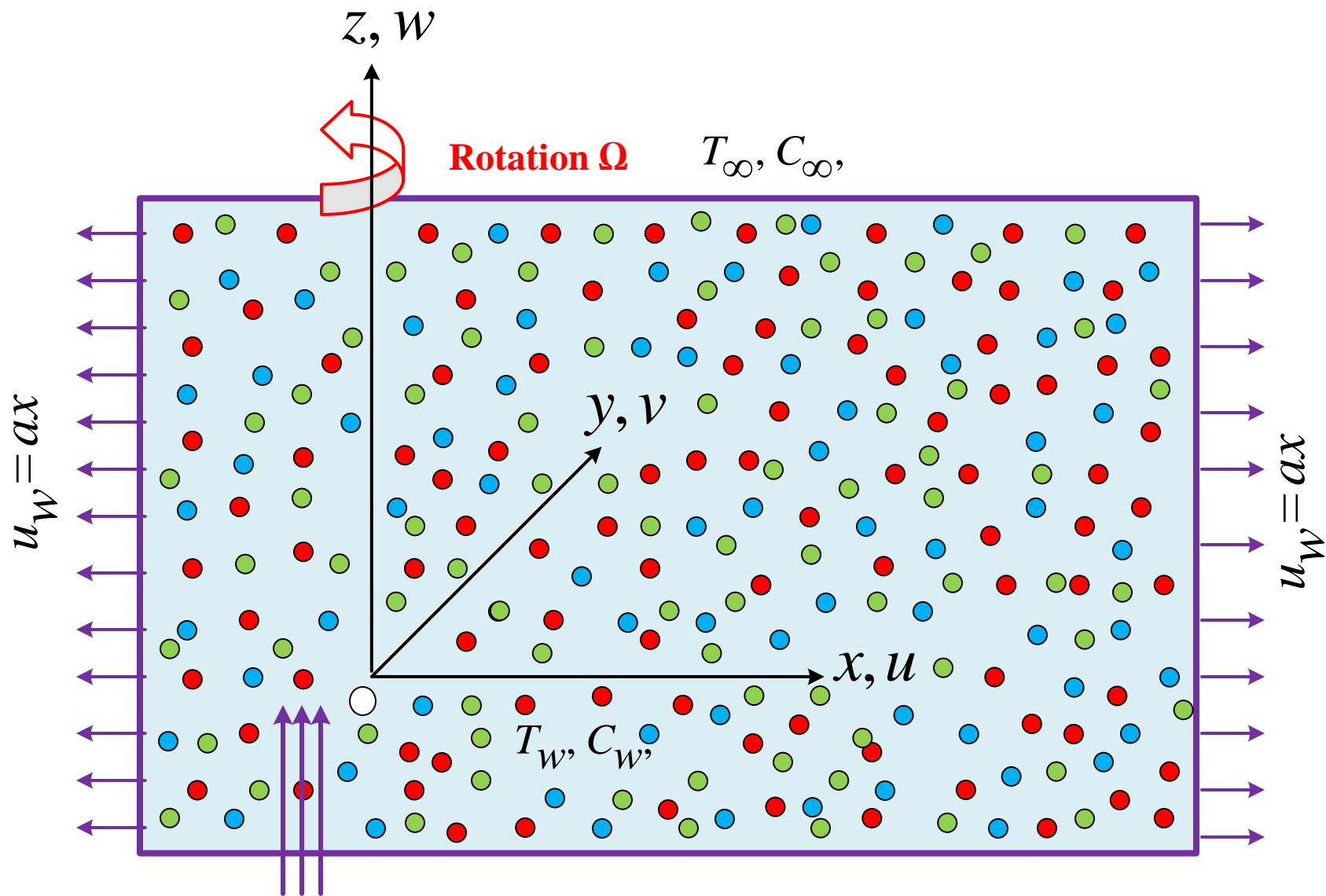

Magnetic field (B<sub>0</sub>), ● Nanoparticles, ● Reactive species ● Porous medium

Supplement: Supplementary file 1 — Supplementary Information. [file 41598_2023_41141_MOESM1_ESM.zip › P22- dusty Ellis tri/Sonia_Model.pdf]

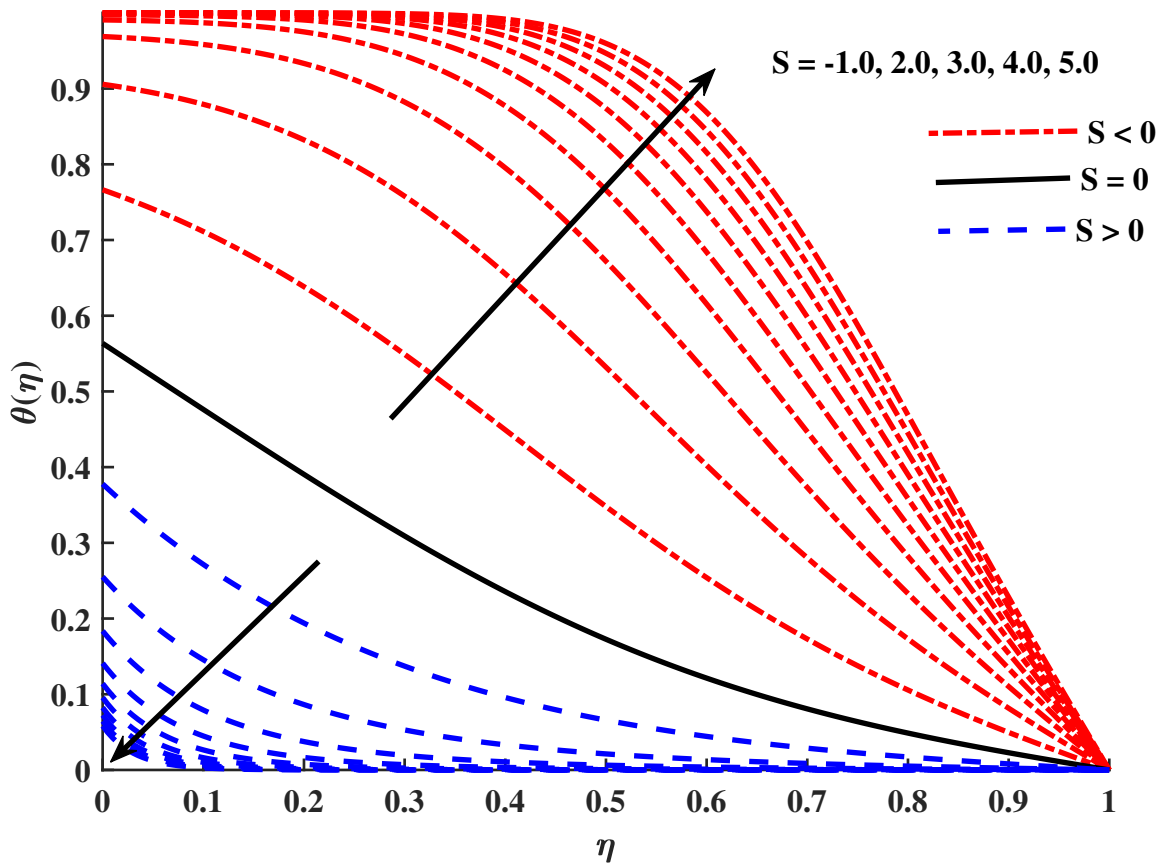

Supplement: Supplementary file 1 — Supplementary Information. [file 41598_2023_41141_MOESM1_ESM.zip › P22- dusty Ellis tri/STheta-eps-converted-to.pdf]
